# Epithelial IL-33 appropriates exosome trafficking for secretion in chronic airway disease

**DOI:** 10.1172/jci.insight.136166

**Published:** 2021-02-22

**Authors:** Ella Katz-Kiriakos, Deborah F. Steinberg, Colin E. Kluender, Omar A. Osorio, Catie Newsom-Stewart, Arjun Baronia, Derek E. Byers, Michael J. Holtzman, Dawn Katafiasz, Kristina L. Bailey, Steven L. Brody, Mark J. Miller, Jennifer Alexander-Brett

**Affiliations:** 1 Department of Medicine, Division of Pulmonary and Critical Care Medicine, and; 2 Department of Cell Biology and Physiology, Washington University School of Medicine, St. Louis, Missouri, USA.; 3 Department of Medicine, Division of Pulmonary, Critical Care, Sleep and Allergy, University of Nebraska Medical Center, Omaha, Nebraska, USA.; 4 Department of Medicine, Division of Infectious Diseases, and; 5 Department of Pathology and Immunology, Washington University School of Medicine, St. Louis, Missouri, USA.

**Keywords:** Immunology, Pulmonology, COPD, Cellular immune response, Cytokines

## Abstract

IL-33 is a key mediator of chronic airway disease driven by type 2 immune pathways, yet the nonclassical secretory mechanism for this cytokine remains undefined. We performed a comprehensive analysis in human airway epithelial cells, which revealed that tonic IL-33 secretion is dependent on the ceramide biosynthetic enzyme neutral sphingomyelinase 2 (nSMase2). IL-33 is cosecreted with exosomes by the nSMase2-regulated multivesicular endosome (MVE) pathway as surface-bound cargo. In support of these findings, human chronic obstructive pulmonary disease (COPD) specimens exhibited increased epithelial expression of the abundantly secreted *IL33*^Δ34^** isoform and augmented nSMase2 expression compared with non-COPD specimens. Using an *Alternaria*-induced airway disease model, we found that the nSMase2 inhibitor GW4869 abrogated both IL-33 and exosome secretion as well as downstream inflammatory pathways. This work elucidates a potentially novel aspect of IL-33 biology that may be targeted for therapeutic benefit in chronic airway diseases driven by type 2 inflammation.

## Graphical abstract

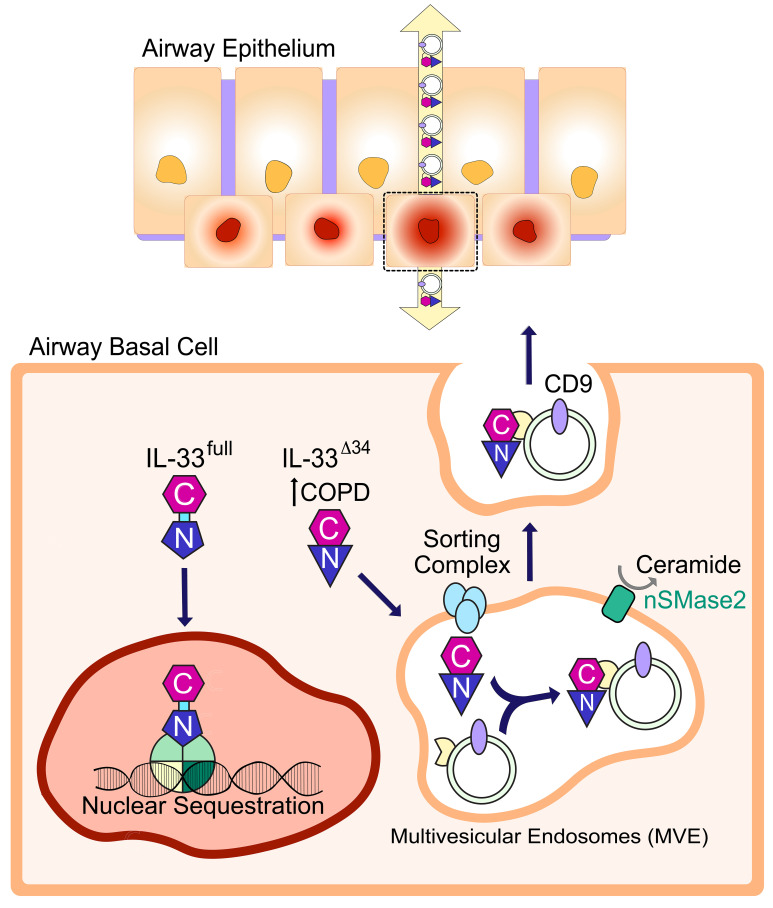

## Introduction

IL-33 is a cytokine abundantly expressed at mucosal barriers (1) that has been shown to promote type 2–polarized immune programs in health and disease (2, 3). A role for IL-33 in human type 2–driven chronic airway disease was highlighted by genome-wide association studies linking *IL33* and *IL1RL1/ST2* with asthma (4–6) and increased IL-33 in serum, sputum, or tissue from patients with asthma (7, 8) and patients with chronic obstructive pulmonary disease (COPD) (9–11). Animal models have also supported a role for IL-33 in response to virus (9) or allergen (12, 13) triggers of airway disease. The IL-33 system is also broadly linked to pulmonary inflammation, arthritis, inflammatory bowel disease, hepatitis, heart failure, central nervous system inflammation, and cancer (reviewed in ref. 2). This wide-ranging disease relevance emphasizes the necessity of understanding how a nuclear-targeted cytokine, or “nucleokine,” lacking a classical secretion signal can be released from intact cells to propagate inflammation.

Human full-length IL-33 (IL-33^full^) is a 270–amino acid protein encoded by 8 exons that encompass the N-terminal “chromatin-interacting” domain (NTD) and C-terminal “cytokine” domain (CTD). The NTD chromatin-interacting motif (14) appears to be responsible for tightly sequestering IL-33 within the nucleus, presumably to regulate the cytokine, as a specific nuclear function for IL-33^full^ protein has yet to be firmly established (15). The CTD includes exons 5–8 and is sufficient to induce signaling through the IL-33 receptor (IL-1RL1/ST2 and IL-1RAP) (16). Unlike the prototypic IL-1 family member, IL-1β, IL-33 is not cleaved by inflammasome-associated caspases (17, 18). Apoptotic and inflammatory proteases can process IL-33^full^ in vitro, which can result in either enhanced function (19) or deactivation (20, 21). While these in vitro studies have shown that processed cytokine retains activity, it remains unknown whether proteolysis is required for activation or secretion of endogenous IL-33 in vivo.

IL-33 secretion has been investigated under multiple conditions, and two primary scenarios have unfolded in the literature (reviewed in ref. 22): passive “alarmin” release from necrotic cells during tissue damage and nonclassical secretion from intact cells. In the context of airway disease, passive IL-33 release could occur with acute airway damage, but this transient activity is unlikely to account for maintenance of chronic disease. A mechanism based on nonclassical secretion appears to be the more likely basis for persistent IL-33 activity. Support for this mechanism is provided by the recent description of natural spliced *IL33* isoforms lacking portions of the NTD (23, 24), which may not be regulated by nuclear sequestration.

To investigate the mechanism of epithelial IL-33 secretion in chronic airway disease, we took advantage of the properties of these truncated *IL33* isoforms. We expressed IL-33 protein variants in primary human airway basal cells as dual-fluorescence and peptide-tagged fusion proteins, demonstrating secretion of intact protein through the neutral sphingomyelinase-2–dependent (nSMase2-dependent) multivesicular endosome (MVE) pathway. IL-33 can be cosecreted noncovalently bound to small extracellular vesicles (EVs) (approximately 100–150 nm diameter) commonly referred to as exosomes (25). We also leveraged COPD specimens to investigate this pathway and found the IL-33 isoform lacking NTD exons 3 and 4 (*IL33*^Δ34^**) and nSMase2 were increased in COPD-derived specimens relative those in non-COPD controls. IL-33 was isolated from COPD bronchial wash (BW) fluid, and the primary species present was a truncated, bioactive form with immunoreactivity consistent with the IL-33^Δ34^ variant. Using an *Alternaria*-induced model of airway disease, we demonstrated that blockade of nSMase2 with GW4869 resulted in reduced IL-33 and exosome secretion in bronchoalveolar lavage (BAL) fluid and downstream type 2 inflammation. Together, these data reveal a mechanism for IL-33 secretion from intact airway cells and demonstrate a potential avenue for the development of novel therapeutics in type 2 endotypes of chronic airway disease driven by this cytokine pathway.

## Results

### Tonic secretion of truncated IL-33 variants from intact airway cells.

**Figure 1:**
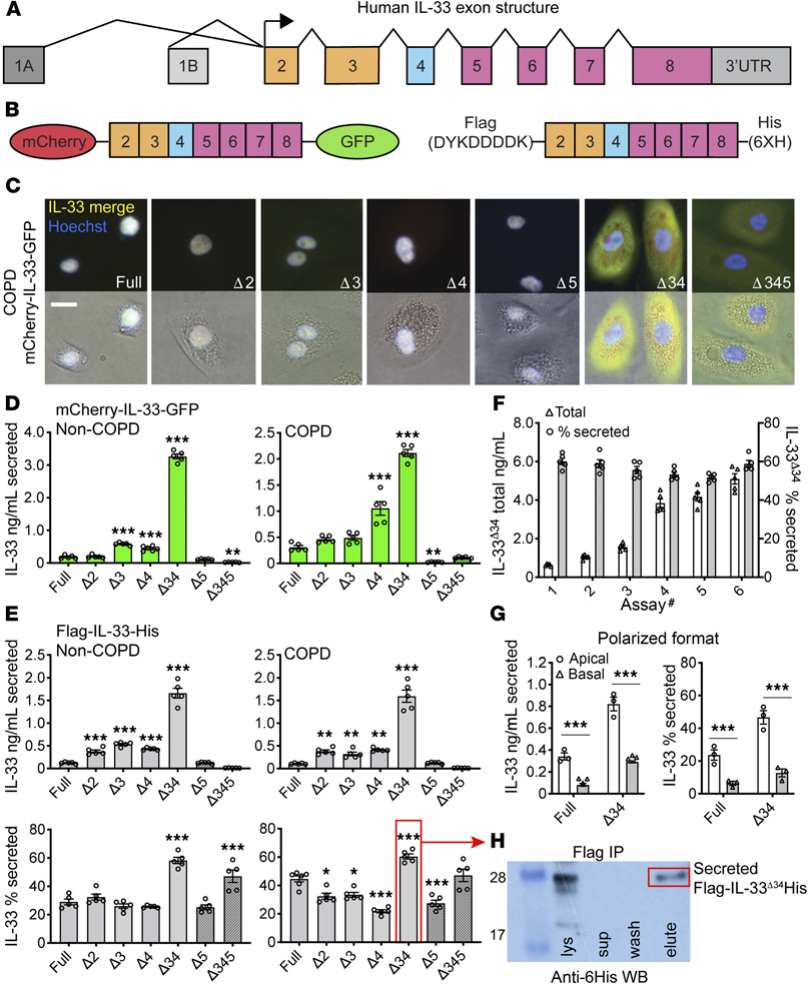
Tonic secretion of IL-33 from airway epithelial cells. (**A**) Human *IL33* exon structure with N-terminal domain (orange), interdomain linker (cyan), and C-terminal domain (magenta). (**B**) Schematic of human IL-33 fusion protein reagents cloned with either dual-fluorescence (N-terminal mCherry and C-terminal GFP) tags or dual-peptide (N-terminal Flag and C-terminal 6xHis) tags. (**C**) Live-cell imaging of COPD airway basal cells transduced with lentiviruses expressing the following mCherry-IL-33-GFP variants: full length (Full) or truncated lacking single exons 2–5 (Δ2, Δ3, Δ4, and Δ5) or multiple exons (Δ34 and Δ345). Cytoplasmic yellow (merged) staining is shown for Δ34 and Δ345 variants; Hoechst 33342 counterstain was also used. Scale bar: 10 μm. (**D** and **E**) ELISA secretion assay performed for mCherry-GFP and Flag-His IL-33 variants in non-COPD and COPD airway basal cells (*n* = 5). Solid colored bars show measurement with R&D monoclonal assay, and bars with angled stripes (Δ5 and Δ345) indicate measurement with polyclonal assay (due to lack of monoclonal epitope located in exon 5). (**F**) Secretion assay for Flag-IL-33^Δ34^-His, demonstrating that the percentage of secretion is stable over a 10-fold expression range in non-COPD cells (*n* = 5). (**G**) Secretion assay in polarized format for non-COPD cells expressing full-length Flag-IL-33^full^-His and truncated Flag-IL-33^Δ34^-His variants (*n* = 3). Protein was detected in both apical and basal fractions, with apical predominance as both concentration and percentage secreted. (**H**) Flag IP of Flag-IL-33^Δ34^-His from COPD cell supernatant. Lanes: cell lysate (lys), supernatant (sup), and Flag-IP supernatant (elute) detected by anti-6His Western blot demonstrating intact (unprocessed) secreted protein. Data are shown as the mean ± SEM. Statistical analysis: 1-way ANOVA (**D** and **E**) and *t* test (**G**); *P < 0.05, **P < 0.01, ***P < 0.001.

We leveraged a series of known N-terminal truncated *IL33* isoforms (23, 24) lacking exons 3–5 (Figure 1A) to investigate the mechanism of nonclassical secretion from intact airway epithelial cells. We transduced airway basal cells with lentiviruses expressing dual-tagged IL-33 fusion proteins, comprising either N-terminal mCherry and C-terminal monomeric eGFP (mCherry-IL-33-GFP) or N-terminal Flag and C-terminal 6-His tags (Flag-IL-33-His) (Figure 1B). This strategy allowed simultaneous tracking of N- and C-terminal fragments, should proteolysis occur in the process of secretion. We imaged live COPD basal cells (Figure 1C) and stable HBE-1 cell lines (Supplemental Figure 1, A and B; supplemental material available online with this article; https://doi.org/10.1172/jci.insight.136166DS1) expressing mCherry-IL-33-GFP fusion proteins, including IL-33^full^, single-exon deletion variants (IL-33^Δ3^, IL-33^Δ4^, IL-33^Δ5^), compound deletion variants (IL-33^Δ34^ and IL-33^Δ345^), and a nonnatural IL-33^Δ2^ variant (for comparison). Results demonstrated tight nuclear mCherry and GFP signal for IL-33^full^ and all single-exon deletion variants. In contrast, the compound deletion variants exhibited mixed nuclear and cytoplasmic staining, as apparent by both epifluorescence (Figure 1C) and confocal imaging (Supplemental Figure 1B). The strict merged signal for all variants indicated that proteins were intact within respective cellular compartments.

To determine whether altered cellular localization affected IL-33 secretion efficiency, we performed IL-33 ELISA on cell supernatants and lysates for epithelial cells expressing IL-33 variants as both mCherry-GFP and Flag-His fusions (Figure 1, D and E, and Supplemental Figure 1, C–E). In both COPD and non-COPD basal cells as well as the epithelial HBE-1 cell line, we found that IL-33^Δ34^ exhibited the most abundant protein expression and tonic secretion among the isoforms tested. When normalized to account for differences in protein expression levels (percentage secreted, Figure 1E and Supplemental Figure 1, C and D), IL-33^Δ34^ was still secreted more efficiently than any other variant, a finding which was stable over a 10-fold range of total protein expression (Figure 1F). The IL-33^Δ345^ variant also exhibited increased secretion efficiency, but total expression was well below other variants and near assay detection limits.

We also tested vectoral secretion of IL-33^full^ and IL-33^Δ34^ from non-COPD basal cells assayed in polarized format (Figure 1G). Flag-IL-33-His secretion was measured in apical and basal compartments of confluent cultures grown on Transwell supports, which showed that IL-33^full^ and IL-33^Δ34^ were secreted in both directions but more abundantly from the apical surface. Similar to the nonpolarized format, the IL-33^Δ34^ variant was secreted more efficiently (2-fold) over IL-33^full^. Results were not influenced by the pore sizes in the Transwell support (Figure 1G and Supplemental Figure 1F).

To investigate the role of proteolysis in tonic secretion, we analyzed Flag-immunoprecipitated (Flag-IP) Flag-IL-33^Δ34^-His from cell supernatants by anti-6His Western blot (Figure 1H). Flag-IP IL-33^Δ34^ protein from supernatant migrated at the same MW as protein from cell lysate and was detected by anti-6His Western blot, indicating that secreted protein was intact (not proteolytically processed). This is consistent with imaging of mCherry-IL-33-GFP isoforms, which demonstrated merged signal in both nuclear and cytoplasmic compartments.

Together, these results demonstrate that the protein product of the natural *IL33*^Δ34^** isoform can be abundantly expressed in airway basal cells, exhibits cytoplasmic accumulation due to lack of nuclear targeting, and is tonically secreted at high levels preferentially from the apical surface without proteolytic processing. These findings support a model in which *IL33* isoforms with altered cellular localization are released from nuclear regulation to undergo tonic secretion from the base of the epithelium predominantly toward the airway lumen.

### Epithelial IL-33 and exosomes are secreted through the nSMase2-dependent MVE pathway.

The protein products of all *IL33* isoforms lack a signal sequence to mediate secretion via the ER/Golgi pathway, therefore the tonic secretion observed in our cellular assay must occur via a nonclassical mechanism. Multiple routes of nonclassical protein secretion have been described, including active transporters (26), cell death–inducing membrane pores (27), plasma membrane shedding (microvesicles), or through incorporation into multivesicular endosomes (MVE) that fuse with the cell surface to release small intraluminal EVs or “exosomes” containing protein and miRNA cargo (28).

**Figure 2:**
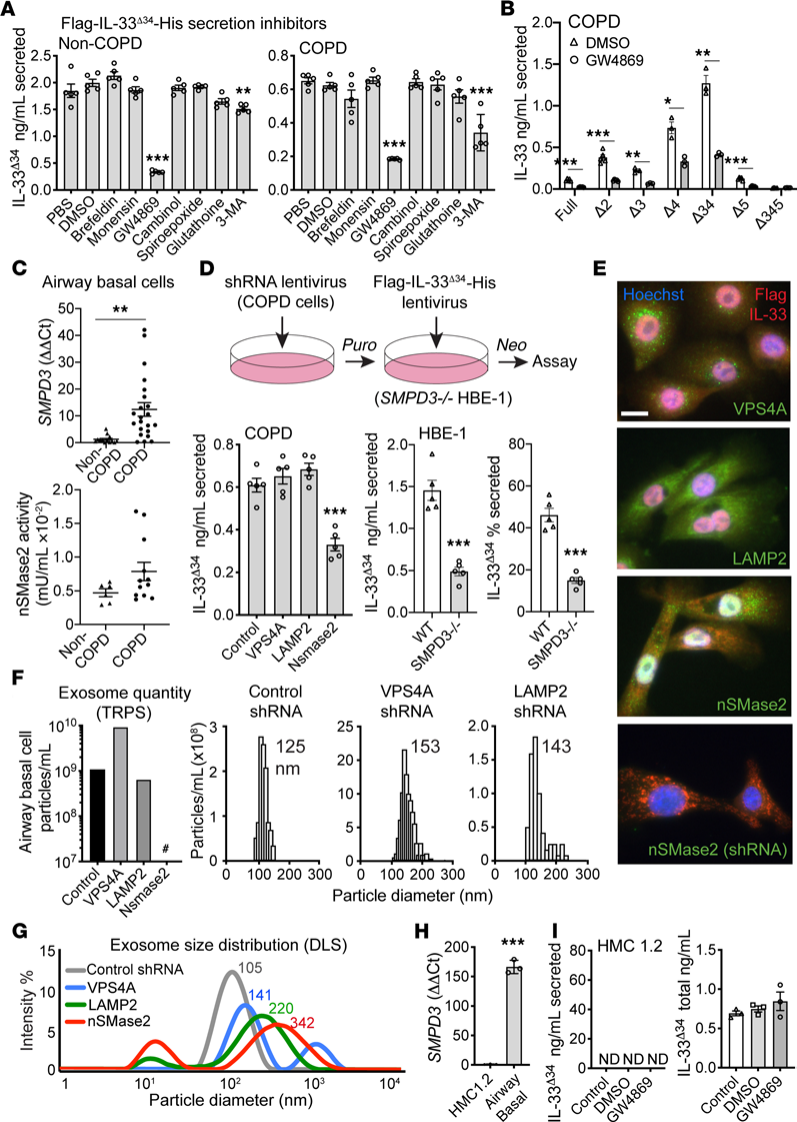
nSMase2 regulates tonic IL-33 and exosome secretion from epithelial cells. (**A**) ELISA secretion assay for Flag-IL-33^Δ34^-His COPD and non-COPD airway basal cells treated with chemical inhibitors (concentrations in Methods), PBS, DMSO vehicle control, and Golgi (brefeldin and monensin); vesicle pathway inhibitors (GW4869, cambinol, spiroepoxide, and glutathione); and microautophagy inhibitors (3-methyladenine [3-MA]), showing marked blockade with GW4869 (*n* = 5). (**B**) COPD cells treated with DMSO or GW4869 (20 μM), demonstrating inhibition for all IL-33 variants (*n* = 3–5). (**C**) qPCR for *SMPD3* mRNA in *n* = 12 non-COPD and *n* = 22 COPD cell specimens and nSMase2 enzyme activity for a subset (*n* = 6 non-COPD, *n* = 12 COPD), demonstrating increased expression in COPD. (**D**) shRNA knockdown in COPD cells and *SMPD3^–/–^* HBE-1 cells, showing decreased Flag-IL-33^Δ34^-His secretion with nSMase2 shRNA, which was further reduced in *SMPD3*^–/–^ HBE-1 cells (*n* = 5). (**E**) COPD cells expressing Flag-IL33^Δ34^-His immunostained for VPS4A, LAMP2, or nSMase2 (green) and Flag (red). Coexpression of nSMase2 shRNA demonstrates loss of staining; Hoechst 33342 nuclear counterstain was also used. Scale bar: 10 μm. (**F**) Secreted exosomes (<150 nm) from shRNA knockdown cells quantified by tunable resistive pulse sensing (TRPS), demonstrating increased particles for VPS4A. ^#^Undetectable for nSMase2 (detection limit 1 × 10^7^ particles/ml). (**G**) Dynamic light scattering (DLS) particle size distribution on same samples with a larger peak shift for nSMase2 shRNA. (**H**) *SMPD3* qPCR demonstrating increased relative expression in airway basal cells compared with HMC1.2 mast cell line (*n* = 3). (**I**) HMC1.2 cells expressing Flag-IL-33^Δ34^-His demonstrate no tonic secretion (ND) despite measurable total protein in lysate (*n* = 3). Data are shown as the mean ± SEM. Statistical analysis: 1-way ANOVA (**A** and COPD in **D**) and *t* test (**B**, **C**, **H**, and HBE-1 in **D**); *P < 0.05, **P < 0.01, ***P < 0.001.

To address which nonclassical secretion pathway was involved, we tested a panel of chemical inhibitors with our IL-33 ELISA secretion assay. We performed inhibition experiments in non-COPD and COPD basal cells and HBE-1 cells expressing Flag-IL-33^Δ34^-His and mCherry-IL-33^Δ34^-GFP (Figure 2A and Supplemental Figure 2, A and B). The ER-Golgi inhibitors brefeldin and monensin had no effect on secretion; however, we did observe a marked inhibition of IL-33^Δ34^ secretion following treatment with GW4869, a noncompetitive antagonist of the ceramide synthetic enzyme neutral sphingomyelinase 2 (nSMase2) (29) (Figure 2A). GW4869 was found to inhibit IL-33^Δ34^ secretion in both non-COPD and COPD basal cells and HBE-1 cells, which was apparent as both the amount and percentage secreted (Figure 2A and Supplemental Figure 2, A and B). Other putative nSMase2 inhibitors, spiroepoxide (29), glutathione (29), and cambinol (30), and the microautophagy inhibitor 3-methyladenine (3-MA) (31) exhibited a modest effect on secretion, which was variably significant among non-COPD, COPD, and HBE-1 cells tested. When GW4869 was tested against the panel of IL-33 variants in COPD cells, it was found to globally inhibit secretion for variants that exhibit distinct cellular localization patterns and expression levels (Figure 2B and Supplemental Figure 2C).

The ceramide biosynthetic enzyme nSMase2 (*SMPD3*) regulates the ESCRT-independent MVE pathway through maturation and membrane fusion of MVEs (29). Our observed inhibition of tonic epithelial IL-33 secretion with the compound GW4869 suggested that this phenomenon may be dependent on nSMase2 activity. We first examined *SMPD3* expression in airway basal cells, which revealed significantly increased expression in COPD specimens relative to that in non-COPD specimens (Figure 2C). nSMase2 enzyme activity was measured in a subset of cultured cell lysates, and multiple COPD specimens exhibited increased activity compared with non-COPD specimens. Given these findings, we targeted the nSMase2-dependent MVE pathway with shRNA knockdown and compared with mediators of the ESCRT-dependent MVE (VPS4A) (32) and microautophagy (LAMP2) (33) pathways. Experiments using primary cells also included the ROCK inhibitor Y27632, which functions to block microvesicle shedding from the plasma membrane (34). In COPD cells, we performed serial rounds of transduction with shRNA- and Flag-IL-33^Δ34^-His–expressing lentiviruses, followed by selection and recovery (Figure 2D). Successful knockdown of targets was confirmed by qPCR (Supplemental Figure 2E). We were careful to fully recover cells before secretion assay to avoid ongoing cell death from selection complicating interpretation. Subsequent IL-33 secretion assay demonstrated a 2-fold reduction in IL-33^Δ34^ secretion only with nSMase2 shRNA knockdown, based on both absolute and percentage secretion (Figure 2D and Supplemental Figure 2D). As a confirmatory approach, we developed an HBE-1 *SMPD3^–/–^* cell line, which revealed further (3-fold) reduction in IL-33^Δ34^ absolute and percentage secretion (Figure 2D and Supplemental Figure 2G). COPD cells expressing Flag-IL-33^Δ34^-His were also immunostained with anti-Flag and corresponding antibodies for VPS4A, LAMP2, and nSMase2, demonstrating a vesicular staining pattern for pathway intermediates and diffuse nucleocytoplasmic staining for IL-33^Δ34^ (Figure 2E). Immunostaining was also performed in cells expressing nSMase2 shRNA and Flag-IL-33^Δ34^-His, which demonstrated a loss of nSMase2 staining and an accumulation of cytoplasmic IL-33^Δ34^ (and loss of nuclear staining).

To investigate the effect of nSMase2 knockdown on EV production, we analyzed EVs secreted from COPD cells (Figure 2F). Culture supernatants were concentrated using a 100 kDa cutoff centrifugal filter, and the concentrations of particles with diameters of less than 150 nm were measured by tunable resistive pulse sensing (TRPS, Figure 2F and Supplemental Figure 3A). Secreted EVs were notably increased 10-fold with VPS4A knockdown but undetectable (<10^7^ particles/ml) for nSMase2 (Figure 2F). Particle size histograms revealed that mean EV size for control cells was 125 nm, consistent with that of exosomes, which was shifted slightly larger with VPS4A or LAMP2 knockdown. Because TRPS analysis is limited to a pre-determined size range, we also analyzed EVs with dynamic light scattering (DLS), which showed that particle histograms shifted to be larger with VPS4A knockdown; this size increase shifted further with LAMP2 knockdown and exceeded exosome size range with nSMase2 knockdown (Figure 2G and Supplemental Figure 3D). For all shRNA knockdown conditions, vesicles exhibited increased heterogeneity based on the polydispersity index compared with control, suggesting that disruption of any pathway intermediate could have global effects on vesicle biogenesis.

We also sought to determine whether this is a general phenomenon, as IL-33 expression has been reported in multiple cell types, including some immune cell populations (13, 35). We found that the human mast cell line HMC 1.2 exhibited 150-fold lower *SMPD3* expression compared with that of airway basal cells (Figure 2H) and, therefore, transduced these cells with Flag-IL-33^Δ34^-His and performed secretion assay. Despite adequate cellular protein expressed in cell lysate, no IL-33^Δ34^ protein could be detected in cell supernatants, including under GW4869 treatment conditions (Figure 2I).

Together, these data reveal that tonic epithelial IL-33^Δ34^ secretion can be blocked by nSmase2 inhibition using both pharmacologic and genetic approaches. Coupled with reduction in secreted exosomes under these same conditions, this strongly implicates the nSMase2-dependent exosome biogenesis pathway in nonclassical IL-33 secretion. Parallel analysis in other cell types further suggests that IL-33 secretion could be a specialized function of nSMase2-expressing cells.

### Augmenting nSMase2 promotes IL-33 secretion.

**Figure 3:**
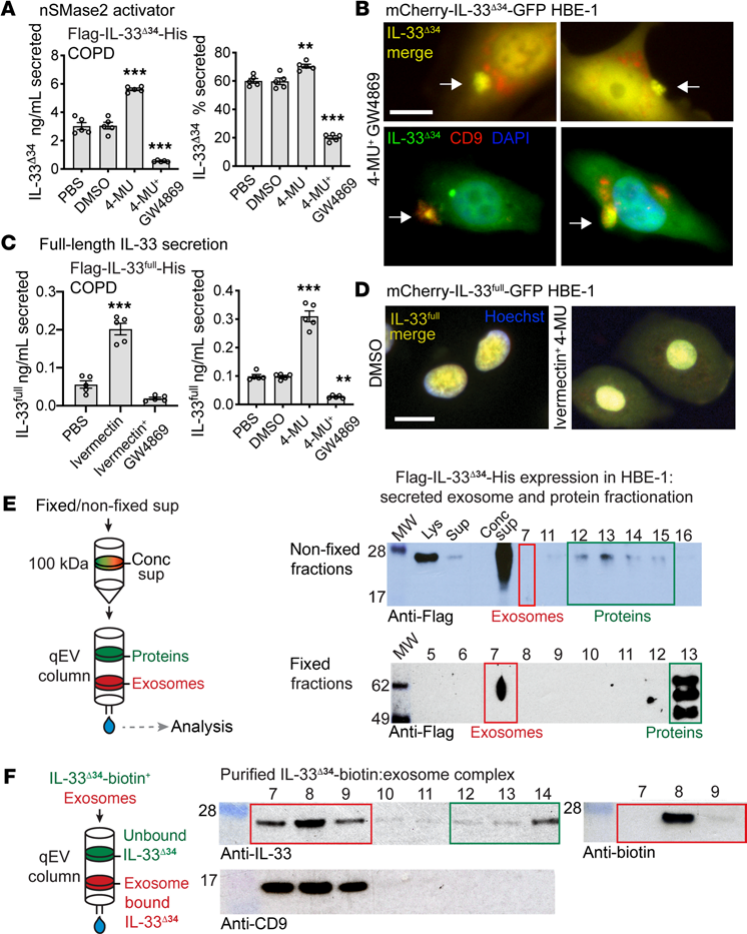
IL-33 cosecretion with exosomes as surface-bound cargo. (**A**) ELISA secretion assay for Flag-IL-33^Δ34^-His expressing COPD airway basal cells treated with nSMase2 activator 4-methylumbelliferone (4-MU, 10 μM) and GW4869 (20 μM). 4-MU augmented secretion, which was blocked by GW4869 (*n* = 5). (**B**) Live-cell imaging for mCherry-IL33^Δ34^-GFP HBE-1 cells treated with 4-MU and GW4869 for 15 minutes, demonstrating foci (white arrows) of yellow merged signal. HBE-1 cells were also fixed and permeabilized after 15 minutes and imaged for GFP only (green) and immunostained for CD9 (red), demonstrating foci of colocalization (white arrows). DAPI nuclear counterstain was also used. Scale bar: 10 μm. (**C**) Secretion of Flag-IL-33^full^-His from COPD airway basal cells was augmented by disruption of nuclear entry (ivermectin, 1 μM) or by nSMase2 activation (4-MU, 10 μM), and both were inhibited by GW4869 (*n* = 5). (**D**) Live-cell imaging of mCherry-IL-33^full^-GFP HBE-1 cells treated with DMSO or ivermectin + 4-MU, showing accumulation of cytoplasmic IL-33^full^ signal within 1 hour. Hoechst 33342 nuclear counterstain was also used. Scale bar: 10 μm. (**E**) Exosome and protein fractionation from Flag-IL33^Δ34^-His–expressing HBE-1 cell supernatant (sup). Secreted IL-33 was retained above centrifugal concentrator 100 kDa filter (conc sup) and detected by anti-Flag Western blot. Lysate (lys) was used for comparison. Exosomes were resolved from free proteins by a qEV size-exclusion column, and fractions were analyzed: nonfixed sup Flag-IL-33^Δ34^-His resolves into protein fractions and fixed sup protein migrates at a higher MW and resolves into both exosome and protein fractions. (**F**) Purified HBE-derived exosomes (10^8^ particles) incubated for 15 minutes with recombinant site–specific biotinylated IL33^Δ34^ protein (1 μg) were resolved on the qEV column, showing that IL-33 coelutes in CD9-containing exosome fractions without fixation. Data are shown as the mean ± SEM. Statistical analysis: 1-way ANOVA (**A** and **C**); *P < 0.05, **P < 0.01, ***P < 0.001.

We used the nSMase2 activator 4-methylumbelliferone (4-MU, ref. 36) to determine whether modulation of nSMase2 activity could promote IL-33^Δ34^ secretion. Treatment of Flag-IL-33^Δ34^-His expressing COPD cells resulted in a 2-fold increase in secreted IL-33^Δ34^ protein and a 10% increase in secretion efficiency, which could be reversed using the noncompetitive nSMase2 inhibitor GW4869 (Figure 3A). Live-cell imaging of the mCherry-IL-33^Δ34^-GFP HBE-1 line treated with both the nSMase2 activator 4-MU and GW4869 in an effort to trap MVEs at the point of secretion demonstrated foci of merged IL-33 signal near the plasma membrane (Figure 3B). Cells were then fixed under the same conditions and immunostained for the exosome marker CD9, which demonstrated that these IL-33-GFP foci also contained CD9.

In contrast to IL-33^Δ34^, IL-33^full^ appears to be tightly sequestered in the nuclei of airway cells. We next asked whether altered cytoplasmic trafficking of IL-33^full^ could drive secretion of this variant through the nSMase2-dependent MVE pathway. We tested this under conditions of nuclear import inhibition with ivermectin (37) and with the nSMase2 activator 4-MU. With ivermectin treatment, we observed an approximately 2-fold increase in secreted IL-33^full^ protein (Figure 3C), which could be reversed by GW4869. Likewise, 4-MU induced a 3-fold enhancement of Flag-IL-33^full^-His secretion, which was also sensitive to GW4869, suggesting that both nuclear import blockade and nSMase2 activation function to shunt cytoplasmic IL-33^full^ toward the MVE secretory pathway. To visualize altered trafficking of IL-33^full^, the mCherry-IL-33^full^-GFP HBE-1 line was treated with both of ivermectin and 4-MU and live-cell imaging was performed, which demonstrated accumulation of cytoplasmic mCherry-IL-33^full^-GFP signal, with sustained signal within the nucleus (Figure 3D). We interpret these results as augmented secretion due to altered trafficking of newly synthesized (overexpressed) IL-33^full^ protein rather than cytoplasmic translocation of nuclear sequestered protein.

These data would suggest that recruitment of IL-33 protein to the nSMase2-dependent MVE pathway could occur for any *IL33* isoform expressed under cellular conditions where the cytokine accumulates within the cytoplasm and that nSMase2 activation can enhance secretion efficiency accordingly.

### *IL-33*^Δ34^ is secreted with exosomes as surface-bound cargo.

As IL-33 and exosome secretion are both sensitive to nSMase2 activity, we next investigated whether IL-33 was in fact associated with exosomes and potentially secreted as cargo upon MVE fusion. We concentrated Flag-IL-33^Δ34^-His–expressing HBE-1 cell supernatant with a 100 kDa centrifugal filter, and Western blot analysis revealed that IL-33^Δ34^ was retained above the filter, even though free protein (MW 25 kDa) would be expected to flow through (Figure 3E). We then resolved exosomes from free proteins by size exclusion chromatography, and Western blot on column fractions revealed that IL-33^Δ34^ signal was absent from exosome fraction 7 but present in free protein fractions 12–15 (Figure 3E). Recognizing that IL-33 could be oxidized in cell culture media (38), we repeated the experiment with fixed, fresh culture supernatant. In fixed supernatant, IL-33^Δ34^ signal was present in exosome fraction 7 but migrated at a higher MW (likely as a result of fixation). We therefore tested whether purified, recombinant IL-33^Δ34^ could bind to separately isolated exosomes. We purified HBE-1 cell–secreted exosomes by size exclusion chromatography and incubated with biotinylated IL-33^Δ34^ protein (Figure 3F). Repeated size exclusion chromatography on the mixture demonstrated clear elution of IL-33^Δ34^ within exosome fractions 7–9, highlighted by CD9, and detected by both anti–IL-33 antibody and streptavidin.

These findings demonstrate that fixation can trap secreted noncovalently bound IL-33^Δ34^ in complex with exosomes in culture supernatant and that exogenously applied IL-33^Δ34^ can form a stable complex with purified exosomes. Collectively, these data illuminate a pathway for IL-33^Δ34^ secretion, with exosomes as surface-bound cargo via the nSMase2-dependent MVE pathway.

### Expression of the *IL33*^Δ34^ isoform in human COPD.

**Figure 4:**
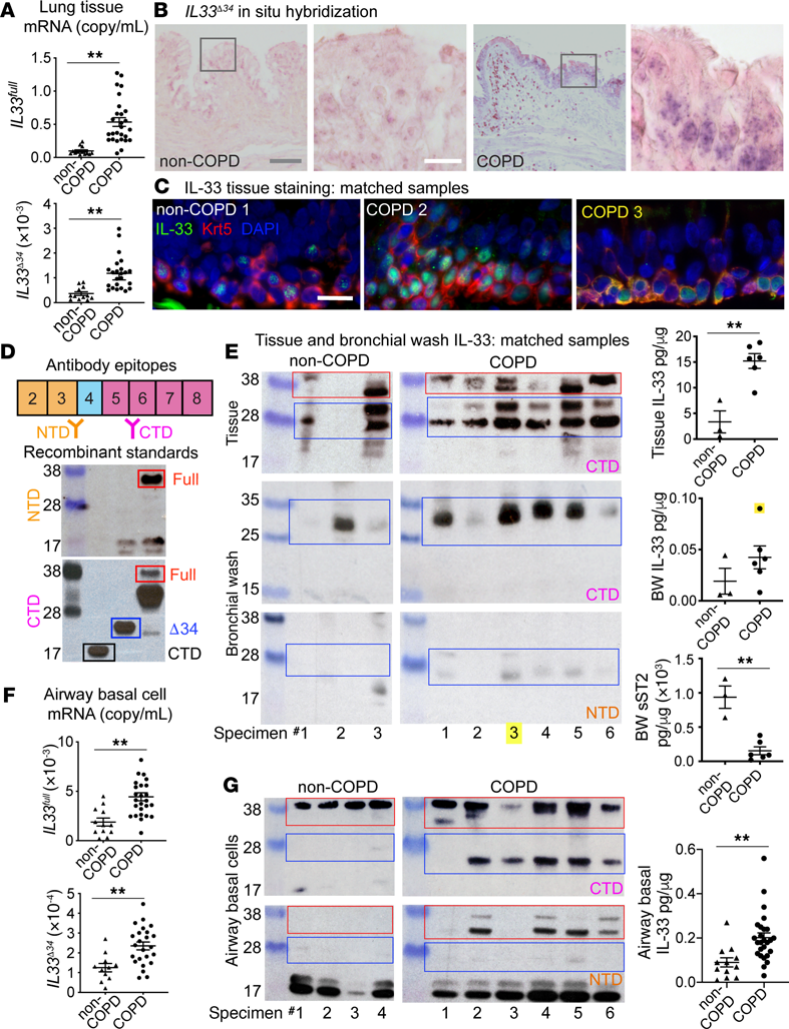
IL33 isoforms in COPD specimens. (**A**) *IL33* isoform–specific qPCR in lung tissue specimens for *IL33^full^* (non-COPD, *n* = 13; COPD, *n* = 27) and *IL33^Δ34^* (non-COPD, *n* = 12; COPD, *n* = 23). (**B**) In situ hybridization in non-COPD and COPD tissues targeting the *IL33^Δ34^* isoform with increased staining in COPD tissue (violet). Nuclear fast red counterstain was also used. Scale bar: 50 μm (gray); 10 μm (white). (**C**) Immunostaining of non-COPD and COPD tissue with IL-33 (green) and cytokeratin 5 (Krt5, red), showing variable cytoplasmic IL-33 staining prominent in COPD 3; DAPI counterstain was also used. Scale bar: 10 μm (white). (**D**) Epitopes for IL-33 NTD and CTD targeting antibodies and reactivity validated with IL-33 variants expressed in HEK293T cells: IL-33^full^ 38 kDa (red, 30 kDa degraded band), IL-33^Δ34^ 28 kDa (blue), and IL-33 CTD 17 kDa (black, Peprotech). (**E**) Matched tissue and bronchial wash (BW) non-COPD (*n* = 3) and COPD (*n* = 6) specimens. Western blot shows multiple fragments detected by CTD antibody in tissue and a 28 kDa band detected by CTD but not NTD antibody in BW. ELISA-quantified IL-33 in the same samples, normalized to total protein. IL-33 protein quantity was increased and soluble ST2 (sST2) was decreased in COPD. COPD 3 with the highest level of BW IL-33 protein is highlighted in yellow. (**F**) *IL33* isoform–specific qPCR in airway basal cells for *IL33^full^* (non-COPD, *n* = 12; COPD, *n* = 26) and *IL33^Δ34^* (non-COPD, *n* = 11; COPD, *n* = 24) (mean ± SEM). (**G**) Western blot on a subset of airway basal cell lysates showing a 28 kDa band detected in COPD by CTD but not NTD antibody. ELISA-quantified normalized IL-33 protein in non-COPD (*n* = 12) and COPD (*n* = 26) cells, showing that IL-33 is increased in COPD. Data are shown as the mean ± SEM. Statistical analysis: *t* test (**A**, **E**, **F**, and **G**); **P < 0.01.

To provide context for our in vitro observations, we examined human COPD tissue specimens for expression of *IL33* isoforms. We analyzed specimens from subjects with very severe COPD undergoing lung transplantation compared with donor lungs unsuitable for transplant (non-COPD) (Supplemental Table 1). Using isoform-specific qPCR assays, we observed that *IL33^full^* and *IL33*^Δ34^** isoforms were significantly increased in COPD tissue, unlike *IL33*^Δ3^**, *IL33*^Δ4^**, and *IL33*^Δ345^** isoforms, which were detected but not significantly upregulated (Figure 4A and Supplemental Figure 4, A and B). To define the expression pattern of *IL33*^Δ34^** in lung tissue, we performed in situ hybridization using an isoform-specific probe (Figure 4B and Supplemental Figure 5A). We found *IL33*^Δ34^** signal to be enriched in cells at the base of the epithelium in COPD compared with non-COPD tissue (Figure 4B and Supplemental Figure 5, C and D), suggesting that airway epithelial basal cells were the primary source of increased *IL33*^Δ34^** transcript. When the same tissue sections were immunostained for IL-33, protein staining could be observed in corresponding regions with high *IL33*^Δ34^** probe staining (Supplemental Figure 5D).

To further examine the protein product of *IL33*^Δ34^** in COPD tissue, we analyzed a subset of non-COPD and COPD specimens for which matched tissue sections, protein lysates, and BW samples were available. We immunostained tissue sections for IL-33 and the basal cell marker cytokeratin 5 (Krt5) in order to highlight the cellular localization of IL-33 protein in COPD airways (Figure 4C and Supplemental Figure 6). Representative non-COPD tissues exhibited lower-intensity, predominantly nuclear IL-33 staining patterns at the base of the airway epithelium. In COPD sections, IL-33 staining exhibited variable patterns, including intense nuclear and diffuse vesicular patterns as well as a strong signal that colocalized with the cytoplasmic basal cell marker Krt5 in one specimen (Figure 4C, COPD 3). We next analyzed these specimens by Western blot to characterize the MW of IL-33 protein products within tissue lysates and BW fluid. We used commercial IL-33 antibodies raised against either NTD (exon 3–4) or CTD (exon 5), which were validated against recombinant IL-33 variants (Figure 4D). Western blot in tissue using the CTD antibody shows multiple variable-intensity bands in MW ranges corresponding to IL-33^full^ and IL-33^Δ34^ in both COPD and non-COPD specimens (Figure 4E). NTD antibody staining from tissue lysates could not be interpreted due to high background signal (data not shown). Parallel analysis of BW samples demonstrated an approximately 28 kDa band of variable intensity detected by the CTD antibody, but not the NTD antibody, suggesting the absence of exon 3–4 epitope (Figure 4E). IL-33 protein was quantified in equivalent tissue and BW samples (normalized to total protein), revealing significantly elevated IL-33 levels in tissue and a trend toward increased levels in BW fluid. Soluble IL-1RL1/ST2 was also quantified and found to be significantly reduced in COPD samples, suggesting a deficiency in soluble receptor–mediated IL-33 neutralization in COPD BW specimens. Among the matched samples in this analysis, COPD 3 was of particular interest, as this specimen demonstrated strong cytoplasmic IL-33 signal in tissue, an intense CTD-reactive 28 kDa band on Western blot, and the highest IL-33 protein level measured in BW by ELISA (highlighted in yellow, Figure 4, C and E).

Analysis of a separate cohort of cultured airway basal cells also revealed increased *IL33^full^* and *IL33*^Δ34^** expression (Figure 4F) compared with that in non-COPD controls. Similar to that in tissue specimens, *IL33*^Δ3^**, *IL33*^Δ4^**, and *IL33*^Δ345^** were detected but not significantly upregulated (Supplemental Figure 4B). Western blot analysis on a subset of these cells using NTD and CTD antibodies as above demonstrates again the presence of an approximately 28 kDa band reactive with CTD antibody but not NTD, which appears enriched in COPD basal cells (Figure 4G). ELISA-quantified IL-33 in the airway cell cohort demonstrates increased total protein in COPD airway cell specimens relative to non-COPD, similar to the trend observed in tissue specimens.

Together, these results provide support for enrichment of the spliced *IL33*^Δ34^** isoform in COPD airway epithelium, which we have found is capable of tonic secretion from basal cells. One COPD specimen demonstrated particularly strong cytoplasmic IL-33 signal in tissues, with concomitant high levels of a truncated protein in BW that exhibited an immunoreactivity profile consistent with the IL33^Δ34^ variant.

### nSMase2 pathway in COPD specimens.

**Figure 5:**
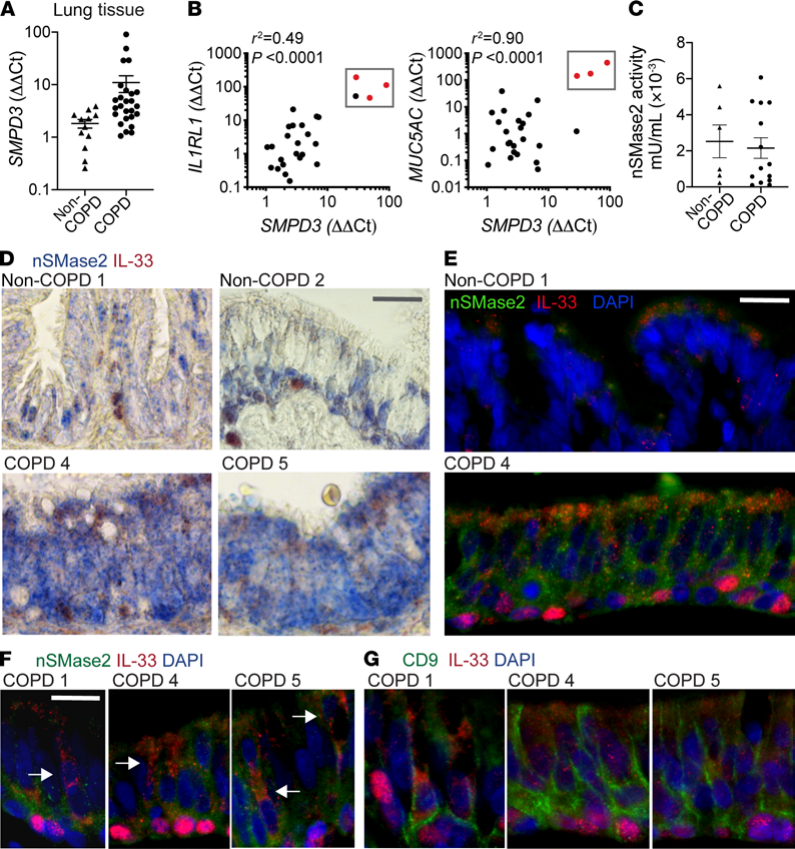
Neutral sphingomyelinase 2 in COPD. (**A**) *SMPD3* qPCR in non-COPD (*n* = 12) and COPD (*n* = 26) lung tissue specimens, demonstrating increased expression in COPD (logarithmic scale due to wide variation in expression level). (**B**) Pearson’s correlation analysis of *SMPD3* versus *IL1RL1* and *MUC5AC* expression. Three specimens exhibited high relative expression in COPD compared with non-COPD specimens for all 3 transcripts (red and boxed). (**C**) Neutral sphingomyelinase 2 (nSMase2) activity in non-COPD (*n* = 6) and COPD (*n* = 14) lung tissue normalized to total protein. (**D**) Immunohistochemistry in non-COPD and COPD tissue stained for nSMase2 (blue) and IL-33 (red); nuclear fast red counterstain was also used. Scale bar: 10 μm. (**E**) Immunofluorescence staining in non-COPD and COPD tissue for nSMase2 (green) and IL-33 (red) with DAPI counterstain. Scale bar: 10 μm. (**D** and **E**) Both methods demonstrate increased nSMase2 signal in COPD airways. (**F**) Focal areas of nSMase2 and IL-33 costaining, with punctate cytoplasmic IL-33 pattern extending toward airway lumen (white arrows). Scale bar: 10 μm. (**G**) Immunostaining for IL-33 (red) with CD9 (green) showing variable IL-33 cytoplasmic signal with surrounding reticular CD9 pattern. Scale bar: 10 μm.

nSMase2 metabolizes sphingomyelin to generate ceramide, and both lung nSMase2 activity and ceramide metabolism have been shown to be altered in the setting of cigarette smoking (39, 40) and COPD (41–43). We have shown that nSMase2 is increased in COPD airway basal cells and regulates tonic IL-33 secretion, and therefore extended the analysis to our cohort of COPD and non-COPD tissue specimens. We found that *SMPD3* exhibited highly variable expression in lung tissue and was increased in multiple COPD specimens, in some by orders of magnitude (Figure 5A). *SMPD3* expression correlated with the IL-33 receptor *IL1RL1* and the major airway mucin upregulated in COPD, *MUC5AC*; in some samples, these 3 transcripts were coincidentally increased by multiple orders of magnitude (Figure 5B, boxed red data points). Though *SMPD3* expression trended higher in COPD tissue samples, this did not translate to a difference in nSMase2 activity observed in a subset of specimens, likely in part due to the 10-fold lower activity in tissue compared with airway cells (Figure 2C and Figure 5C). Immunohistochemistry and immunofluorescence staining of nSMase2 and IL-33 in tissue sections demonstrated a patchy basilar pattern in non-COPD tissue and a more intense, diffuse staining pattern in COPD tissues (Figure 5, D and E, and Supplemental Figure 7, A and B). Frequently nSMase2-enriched epithelium was coincident with cells exhibiting a vesicular cytoplasmic IL-33 pattern that extended toward the airway lumen; examples for multiple COPD specimens are shown in Figure 5F. These regions were also stained for the exosome marker CD9 (separately due to same antibody host species), which demonstrated a striking linear-reticular pattern surrounding IL-33^+^ basal cells extending to the subepithelial and luminal surfaces; examples are shown in Figure 5G.

Together, these data reveal that *SMPD3* expression and nSMase2 protein staining are enriched in COPD specimens, and, in some cases, expression was strongly induced concomitant with *IL1RL1* and *Muc5AC*. nSMase2 and CD9 staining in proximity to cells exhibiting cytoplasmic and/or vesicular IL-33 staining patterns suggests the appropriate machinery is in place to facilitate secretion of IL-33–exosome complexes into the airway lumen and interstitium.

### Isolation of IL-33 and exosomes from COPD BW.

**Figure 6:**
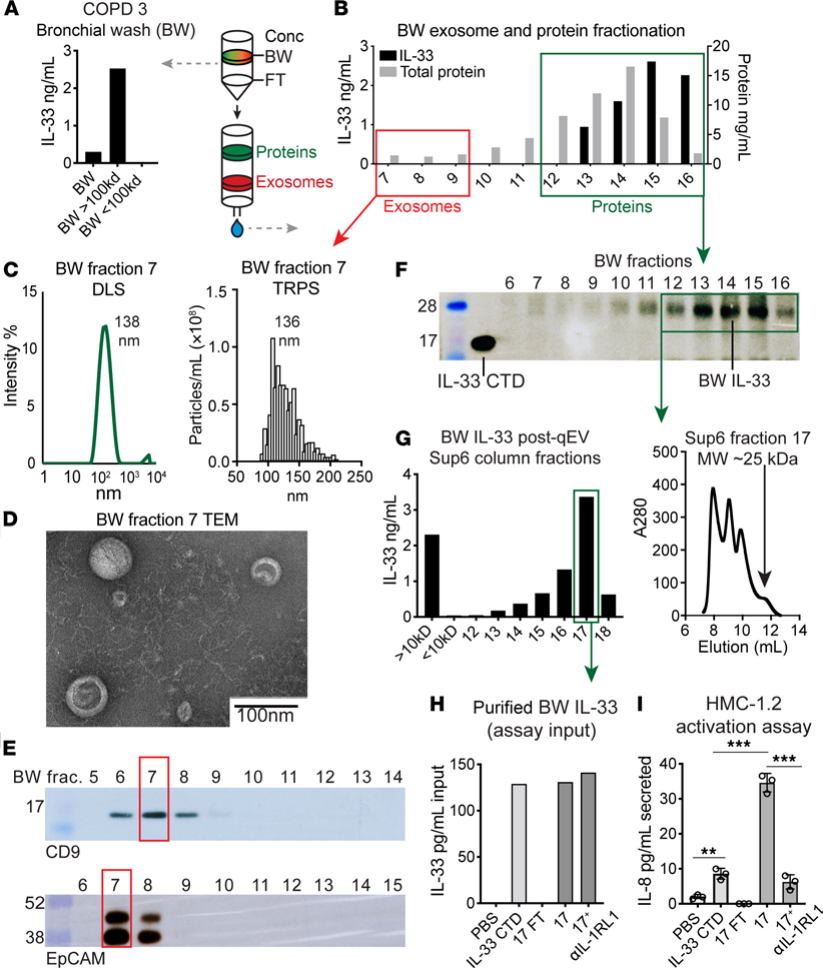
Analysis of IL-33 and exosomes from COPD bronchial wash specimens. (**A**) ELISA quantified IL-33 in concentrated bronchial wash (BW) fluid (100 kDa filter cutoff) with retention of endogenous IL-33 above the filter. (**B**) IL-33 and protein concentration measured in fractions eluted from the size-exclusion column (Izon qEV). (**C** and **D**) Extracellular vesicles isolated from BW were consistent with exosomes based on transmission electron microscopy (TEM), tunable resistive pulse sensing (TRPS), and dynamic light scattering (DLS). Scale bar: 100 nm. (**E**) Western blot with CD9^+^ and EpCAM^+^ exosome fractions, indicating an epithelial source. (**F**) Western blot (CTD antibody) showing elution of truncated IL-33 protein (MW approximately 28 kDa), similar to that shown in Figure 4E. See the IL-33 CTD fragment for reference. (**G**) ELISA analysis of qEV fractions subject to further resolution with Superose6 (Sup6) size exclusion chromatography. Elution profile with peak IL-33 in fraction 17 corresponds to MW = 25 kDa based on protein standard curve. (**H** and **I**) HMC1.2 activation assay for purified endogenous BW IL-33 protein. Input IL-33 concentration for purified protein and CTD standard verified by ELISA due to low BW IL-33 yield. IL-8 level in cell supernatant measured by ELISA after 12 hours of incubation with CTD standard, concentrator flow through (FT, negative control), or endogenous IL-33 with or without IL-1RL1 blocking antibody (100 ng/ml, Proteintech). Data are shown as the mean ± SEM. Statistical analysis: 1-way ANOVA (**I**); ***P < 0.001.

In parallel with our in vitro analysis of IL-33 and exosomes secreted from cultured airway basal cells, we sought to isolate and characterize endogenous components from BW specimens. We performed this analysis with COPD 3 exhibiting the highest IL-33 level, first by concentrating the BW sample using a centrifugal 100 kDa filter, as in Figure 3E. Similar to culture supernatant, endogenous BW IL-33 was retained above the filter, as quantified in Figure 6A. Concentrated BW was fractionated by size exclusion chromatography, and ELISA-quantified IL-33 as well as total protein levels are shown in Figure 6B. Peak exosome fraction 7 was analyzed by TRPS and DLS (Figure 6C and Supplemental Figure 3, A and B), yielding vesicle size and distribution nearly identical to those of airway cell–derived exosomes, and ultimately confirmed by transmission electron microscopy (Figure 6D). Exosome properties were also consistent for multiple COPD specimens evaluated (Supplemental Figure 3, B and D). Western blot was performed on BW exosome fraction 7 to verify CD9 positivity and epithelial origin based on EpCAM staining (Figure 6E). BW IL-33 largely segregated from exosomes into free protein fractions, as observed for nonfixed airway cell supernatant. Western blot using CTD antibody (Figure 6F) confirmed the approximately 28 kDa band observed in Figure 4E. IL-33-containing fractions were again concentrated (10 kDa filter) and further resolved on a high-resolution Superose 6 size-exclusion column. Endogenous IL-33 eluted at a volume corresponding to MW 25 kDa based on standard curve (Figure 6G), consistent with the calculated MW of IL-33^Δ34^. Purified endogenous BW IL-33 was then tested for bioactivity in an HMC 1.2 activation assay (Figure 6, H and I). Due to limited quantities of purified BW IL-33, samples and controls were carefully matched for input concentration before assay (Figure 6H). Endogenous BW-derived IL-33 was found to induce IL-8 secretion from HMC 1.2 cells with greater potency than commercial (CTD) protein at the same input concentration (125 pg/ml), which was inhibited by anti–IL-1RL1 blocking antibody, demonstrating specificity (Figure 6I).

These results collectively reveal that endogenous IL-33 protein isolated from COPD BW fluid exhibits biochemical properties consistent with IL-33^Δ34^ and retains bioactivity. Furthermore, endogenous IL-33 was retained with higher-MW species during concentration of BW fluid, similar to IL-33^Δ34^ secreted from airway cells. Exosomes derived from COPD BW specimens also demonstrate a marker profile consistent with epithelial origin, suggesting that the bulk of exosomes secreted into COPD airway surface liquid are epithelial derived.

### nSMase2 inhibitor blocks IL-33 secretion and type 2 inflammation in vivo.

We have uncovered a mechanism for tonic IL-33 secretion from human airway cells in vitro and found support for this model in human COPD specimens. To test whether blockade of nSMase2 activity could disrupt IL-33–mediated inflammation in vivo, we employed an allergic airway disease model using the fungal allergen *Alternaria alternata*. We selected the *Alternaria* model for this analysis because it is dependent on IL-33 for induction of type 2 inflammation (44–46) and it robustly induces IL-33 protein in BAL fluid (45). Regarding the strategy for nSMase2 blockade, the spontaneously derived mouse *Smpd3* mutation characterized as *fragilitas ossium* (*fro*) confers severe developmental abnormalities in mice, including osteogenesis imperfecta and high perinatal mortality (47). We therefore chose to focus our efforts on the GW4869 compound that was effective in our in vitro studies and has been successfully applied to other inflammatory mouse models (48, 49).

**Figure 7:**
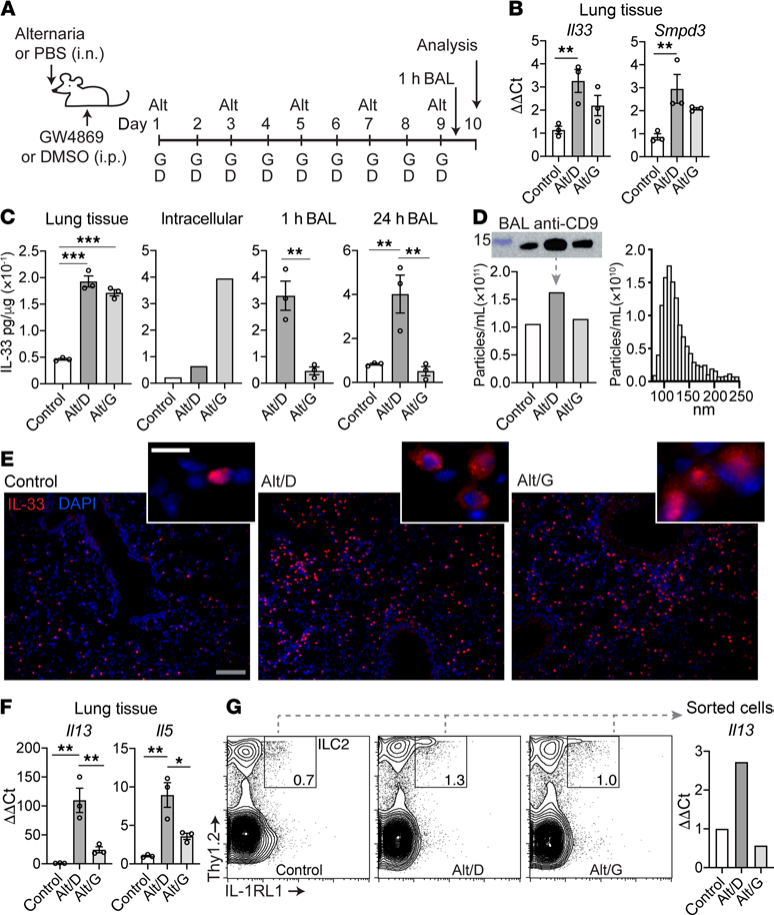
nSMase2 inhibition in *Alternaria* airway disease model. (**A**) Schematic of *Alternaria* experimental model. Mice were treated i.n. with 25 μg *Alternaria* extract (Alt) or PBS (control) every other day for 9 days. Mice receiving Alt were either treated i.p. with DMSO/saline vehicle control (Alt/D) or GW4869 (Alt/G, 2–2.5 μg/g) daily for 9 days. Some mice were analyzed 1 hour following the last Alt dose; the remainder were analyzed 24 hours after the last dose. (**B**) Lung tissue qPCR for the 3 groups (*n* = 3), demonstrating increased *Il33* and *Smpd3* mRNA with Alt treatment. (**C**) IL-33 protein quantified by ELISA (normalized to total protein) for lung lysate (*n* = 3), intracellular fraction (pooled single-cell suspension, *n* = 3 each), and 1-hour (*n* = 3) or 24-hour (*n* = 3) bronchoalveolar lavage (BAL) samples. IL-33 was increased in the intracellular fraction and decreased in BAL with GW4869 treatment. (**D**) Exosome quantity measured by TRPS for pooled (*n* = 3) BAL samples with vesicle quantity, representative size distribution (with peak), and CD9 Western blot, reflecting the decrease in BAL vesicle quantity and corresponding CD9 staining with GW4869 treatment. (**E**) Tissue IL-33 immunofluorescence staining (red) demonstrating increased cytoplasmic IL-33 signal (insets) and expansion of IL-33^+^ parenchymal cells in Alt groups. DAPI counterstain was also used. Scale bar: 50 μm (gray); 10 μm (white). (**F**) Lung tissue *Il13* and *Il5* qPCR (*n* = 3) demonstrating induction with Alt treatment that was attenuated with GW4869. (**G**) Representative FACS plots for sorted lung innate lymphoid type 2 cells (ILC2, pooled samples, *n* = 3) and *Il13* qPCR in sorted cells, demonstrating induction of ILC2 cells and IL13 expression with Alt treatment and concomitant reduction with GW4869 treatment. Data are shown as the mean ± SEM. Statistical analysis: 1-way ANOVA (**B**, **C**, and **F**); *P < 0.05, **P < 0.01, ***P < 0.001.

To induce type 2–driven airway disease in mice, we administered 5 doses of *Alternaria* extract (or PBS) i.n. to mice on alternating days, as an extension of published protocols (44), and treated mice with either GW4869 (Alt/G) or vehicle control (Alt/D) i.p., beginning with the first dose of *Alternaria* and administering daily thereafter (Figure 7A). At 10 days after *Alternaria* treatment, lung *Il33* and *Smpd3* mRNA were found to be increased 3-fold in the Alt/D groups and were not significantly reduced with GW4869 treatment (Figure 7B). Induction was limited to *Il33*, as other type 2 cytokines were not affected by *Alternaria* (*Tslp* unchanged, Supplemental Figure 8C, and *Il25* not detected). Measurement of IL-33 protein revealed a 3-fold induction of total IL-33 in lung tissue for both Alt/D and Alt/G groups (Figure 7C). In contrast, GW4869 appeared to increase intracellular IL-33 protein levels 4-fold in cell suspension, suggesting retention of IL-33 in the setting of nSMase2 blockade. Likewise, IL-33 protein was detected in BAL fluid in the Alt/D group at 1 hour and 24 hours following the fifth dose of *Alternaria* and was markedly decreased in the Alt/G group at both time points. Absolute BAL IL-33 protein was approximately 6-fold higher in BAL at the 1-hour versus 24-hour time points (600 pg/ml vs. 100 pg/ml, respectively), which is not reflected by normalized levels due to the high BAL protein content immediately following the *Alternaria* dose. We attribute induced BAL IL-33 protein to secretion rather than cellular necrosis, as the epithelium appears intact at 1 hour (Supplemental Figure 8B) and 24 hours (Supplemental Figure 8A) after *Alternaria* treatment. Likewise, LDH activity in BAL fluid is marginal compared with that in tissue lysate in samples at 1 and 24 hours, with no observed difference between *Alternaria* and control groups (Supplemental Figure 8B).

Exosomes isolated from BAL fluid 24 hours after the last *Alternaria* dose were analyzed by TRPS and Western blot. Results for pooled replicates showed that the Alt/D group exhibits increased CD9 signal by Western blot and particle number by TRPS, which was decreased to control level with GW4869 treatment. Exosome distribution and mean particle size for the Alt/D group were similar to human airway epithelial and BW derived exosomes (Figure 7D).

IL-33 immunostaining of tissue sections demonstrates *Alternaria*-induced expansion of IL-33^+^ parenchymal cells with alveolar type 2 morphology (Figure 7E), similar to observations in other IL-33–dependent airway disease models (9). This effect was observed in both Alt/D and Alt/G groups, consistent with GW4869 mediating blockade of IL-33 secretion rather than expression. In control tissue, IL-33 exhibited a predominant nuclear pattern, whereas in the Alt/D and Alt/G groups, many cells exhibited a mixed nucleocytoplasmic IL-33 staining pattern (Figure 7E).

With respect to IL-33–induced type 2 inflammation, we analyzed the model at the 6- and 10-day time points and found a 15-fold induction of lung *Il13* mRNA at 6 days (3 doses) and 100-fold at 10 days (5 doses) (Figure 7F and Supplemental Figure 8C). Subsequent analyses were therefore performed at the 10-day time point. In addition to *Il13*, lung *Il5* was also increased 5-fold, and both were substantially decreased with GW4869 treatment. Likewise, lungs sorted for IL-1RL1^+^/Thy1.2^+^ innate lymphoid type 2 (ILC2) cells demonstrated induction of ILC2s in the Alt/D group, which was partially blocked in the Alt/G group (Figure 7G). Analysis of *Il13* expression in sorted cells from pooled replicates demonstrates a 2-fold increase in Alt/D group that was reduced to control level in Alt/G group. We observed a mild increase in *Muc5ac* 6 days after *Alternaria* treatment, which was further induced at 10 days and was augmented with GW4869 treatment (Supplemental Figure 8C). While qualitatively PAS staining appeared diminished in Alt/G tissue sections (Supplemental Figure 8A), it is possible that other IL-33–independent pathways contributing to mucus production are induced in this model (50).

Together, these findings support our model developed in the human system implicating the nSMase2-dependent exosome biogenesis pathway in lung epithelial IL-33 secretion. Disruption of type 2 inflammation in this model is not based on a substantial reduction in *Il33* expression, but rather inhibition of IL-33 secretion, implicating a potentially novel pathway in IL-33–induced chronic airway disease, which we have shown to be amenable to therapeutic intervention.

## Discussion

This study addresses multiple fundamental questions in the field of IL-33 biology and advances our understanding of human chronic airway disease pathogenesis. We show that the IL-33^Δ34^ isoform is increased in COPD and can undergo tonic secretion from airway cells independent of proteolytic processing. We found that this mechanism of secretion from intact airway cells is applicable to all IL-33 variants and occurs through the nSMase2-dependent MVE pathway. Remarkably, IL-33 appears to be secreted as a surface-bound rather than an encapsulated exosome cargo. Identification of nSMase2 as a key mediator of IL-33 secretion and demonstration of increased nSMase2 expression in COPD specimens provides a connection between environmental triggers and nonclassical inflammatory cytokine secretion. We furthermore highlight a therapeutic angle for disrupting the IL-33 system by demonstrating that nSMase2 inhibition can block IL-33 secretion and subsequent type 2 inflammation in a mouse model of airway disease.

Our investigation revealed that tonic secretion of the IL-33^Δ34^ isoform is dependent on the exosome biogenesis pathways regulated by nSMase2. In the context of prior work, multiple stimuli for IL-33 release have been described based on the alarmin hypothesis, including cryoshock (51), proteases (19), respiratory viral infection (52, 53), and allergens, including house dust mite (54). Comparatively, *Alternaria* extract has been reported to induce alarmin release via cellular necrosis (55) or regulated secretion involving purinergic receptors, intracellular calcium signaling, and reactive oxygen species (56, 57). We have also observed modest amounts of endogenous IL-33 secreted from intact airway basal cells exposed to exogenous ATP (9). One common theme among these stimuli is that they all can induce EV flux as a cellular prosurvival signal (58). Therefore, it is possible that a diverse array of cellular signals could converge upon a fundamental cellular pathway that mediates nonclassical protein and exosome secretion. In this case, it appears such a pathway is co-opted by dysregulated *IL33* isoforms expressed in chronic airway disease. It is our expectation that IL-33 would not be the only inflammatory mediator to utilize such a secretory mechanism, indeed this may be a shared phenomenon among IL-1 family members (59, 60). Future studies of nonclassical cytokine secretion will be greatly informed by a more thorough understanding of functional compensation between ESCRT-dependent and -independent MVE pathways and their intersection with chaperone-mediated microautophagy.

The results presented here provide key mechanistic insights into a potential role for EV-mediated cellular communication in chronic airway disease pathogenesis. Indeed, exosome biogenesis pathways have previously been linked to chronic lung disease (61) and nSMase2 has been associated with eosinophilic asthma (62) and found to be increased along with ceramide metabolites in smokers (43) and COPD (42). We have found that nSMase2 is increased COPD specimens and propose a model by which nSMase2 regulates secretion of IL-33 as surface-bound exosome cargo. It will be important going forward to define the molecular interactions between cytoplasmic IL-33 and chaperones that recruit this cytokine to the MVE pathway. Likewise, it will be necessary to determine what role exosome-bound IL-33 plays in the efficiency of IL-33 receptor signaling on effector cells. Future studies should also address whether exosomes can enhance IL-33 stability, given the propensity of this cytokine to be inactivated by protease digestion or oxidation in the extracellular milieu (38).

With respect to the endogenous form of IL-33, our analysis of COPD BW IL-33 protein indicates that a truncated bioactive form is present, with an antibody reactivity profile consistent with the IL-33^Δ34^ isoform. Several prior studies have examined the issue of IL-33 processing, mostly in the context of IL-33^full^ protein. The flexible linker region encoded by exon 4 is not a substrate of caspase-1, in contrast to IL-1β and IL-18 (17, 19, 20), but is sensitive to multiple inflammatory proteases. Given that the secreted IL-33^Δ34^ isoform lacks the protease-sensitive exon 4 linker, it is not surprising that we observe the protein to be secreted in an intact form. Definitive characterization of endogenous IL-33 will require mass spectrometry–based analysis of sufficient quantities of highly purified (nondegraded) protein from relevant biological specimens, which remains a challenging endeavor.

Regarding the limitations of our approach, we understand that our in vitro observations have been made in the context of protein overexpression, which has limitations and could produce unexpected artifacts in any system. We have conducted our experiments carefully to address the phenomenon with different tagged formats and diseased/nondiseased primary cells and cell lines and under a range of expression levels and culture formats to address any irregularities that may result from these experimental conditions.

With respect to our in vivo model, we recognize that COPD is a heterogeneous disorder with multiple described inflammatory endotypes (63), among them type 2 predominant asthma-COPD overlap syndrome (64). Clear challenges remain in defining and validating these endotypes in research and clinical care. In choosing a model system for this study, we considered factors, including variable severity of phenotype (smoking), strain and/or pathogen-specific responses (virus), and distinct respiratory anatomy in mice and humans (IL-33 expression in type 2 pneumocytes in mice vs. basal cells in humans). Multiple studies to date have implicated IL-33 in *Alternaria*-induced respiratory disease and have identified this fungal allergen as a potent stimulus for IL-33 secretion in BAL fluid in vivo. With this in mind, we established the *Alternaria* model to test respiratory IL-33 secretion and downstream inflammation with disruption of nSmase2 activity. We recognize that this model does not encompass the full spectrum of COPD disease.

Clinical COPD care would indeed benefit from a better understanding of which patients may respond more favorably to therapies targeting specific inflammatory endotypes. Our COPD cohort includes sufficient material to explore disease mechanism but is composed of severe COPD specimens, which limits our ability to correlate IL-33^Δ34^ or nSMase2 with COPD disease severity or expression-based metrics of type 2 endotypes (65). Future investigation and validation of pathways illuminated in this study will require the addition of specimens across the spectrum of disease severity and endotypes that incorporate protein- and exosome-focused sampling methods.

In summary, our analysis in human COPD illuminates a role for nSMase2 and exosome pathways in the mechanism of IL-33 airway secretion, supported by amelioration of type 2 inflammation with pharmacological blockade of nSMase2 in vivo. This work reveals a potentially novel aspect of IL-33 biology with the potential to open a new area of investigation in chronic airway disease and development of COPD therapeutics.

## Methods

Additional details are provided in the Supplemental Methods. Details for all materials and reagents used in this study are provided in Supplemental Table 2.

### Human lung samples and study design.

Clinical samples were obtained from consenting patients at the time of lung transplantation from COPD recipients (*n* = 27) with very severe disease (GOLD stage IV) during the period from 2011 to 2019 at Barnes-Jewish Hospital. Control samples were obtained from non-COPD donor lungs (*n* = 13) that were not usable for transplantation at Barnes-Jewish Hospital and University of Nebraska Medical Center. There were no predetermined inclusion or exclusion criteria beyond criteria for lung transplant candidacy. To analyze tissue staining, gene expression, and protein levels, lung tissue samples were collected and processed for histopathology and RNA and protein analysis from 4 different lung zones of each specimen. For this study, equivalent quantities of the 4 lung areas were pooled for RNA and protein analysis to represent a single sample per specimen. Tissue was homogenized in TRIzol (Invitrogen) for all COPD (*n* = 27) and non-COPD (*n* = 13) specimens for RNA extraction. COPD (*n* = 14) and non-COPD (*n* = 6) specimens were minced and lysed in T-PER (Pierce) supplemented with HALT protease inhibitor (Pierce) and centrifuged at 10,000*g*, and supernatant collected for protein analysis. COPD (*n* = 14) and non-COPD (*n* = 6) tissue specimens were fixed in 10% neutral buffered formalin (Thermo Fisher) before paraffin embedding and sectioning for histopathology analysis. Airway basal cells were cultured from large airways (first to third generation) for COPD (*n* = 26) and non-COPD (*n* = 12) specimens, and cells were processed for RNA and protein analysis. For a subset of COPD (*n* = 6) and non-COPD (*n* = 3) specimens, BW was also performed at the time of specimen collection above. BW fluid was obtained from explanted lungs by gently injecting 100 ml PBS into mainstem bronchi, and fluid was recovered with passive return and gentle suctioning (to preferentially return airway surface liquid and minimize alveolar lavage). BW fluid was centrifuged at 100*g* to pellet cells, and HALT was added to supernatant before storage for further analysis.

### Exosome preparations and analysis.

EVs were isolated from BW fluid and cell culture supernatants in an analogous manner. Solutions were first spun at 2000*g* to clarify, followed by concentration using a centrifugal concentrator with 100 kDa MW cutoff (Sartorius Vivaspin Turbo). For low-abundance samples (mouse BAL fluid, shRNA knockdown), samples of equivalent volumes were analyzed following the concentration step. For samples of larger quantity (culture supernatants), equivalent volumes of supernatant from confluent cultures were concentrated and fractionated on a qEV 35 (Izon Science) size-exclusion column. Fractions were collected in PBS supplemented with HALT protease inhibitor and screened by DLS (Malvern NanoS) to verify vesicle size and homogeneity (polydispersity index). Exosomes (100–150 nm) typically eluted in fraction 7–8 and free proteins in fractions 12–17 when run according to manufacturer protocol. For experiments conducted under fixed conditions, following clarification supernatant was fixed with 1% paraformaldehyde for 5 minutes at room temperature and quenched with 1 M Tris pH 8.0. Supernatant was then concentrated similar to the nonfixed sample and exosomes were purified using the qEV 35 column; exosome integrity (size, homogeneity) was verified post-fixation. For purified exosomes mixed with recombinant biotinylated IL-33^Δ34^ protein, approximately 1 × 10^8^ HBE-derived exosomes were incubated with 1 μg IL-33^Δ34^-biotin in 100 μl PBS for 15 minutes at room temperature. Samples were then purified on a qEV 35 column as above.

Exosome analysis was performed using a combination of DLS, transmission electron microscopy, and TRPS. Human and mouse exosomes were typically concentrated 10-fold and quantified by TRPS (Izon Biosciences qNano device) using a 150 nm cutoff pore filter (NP150) and by DLS (Malvern NanoS). Human COPD 3 BAL-derived exosomes were purified by qEV 35 column as above and prepared per ref. 66 for imaging on a JEOL JEM-1400 120 kV transmission electron microscope with an Advance Microscopy Technologies camera system.

### IL-33 secretion assays.

Primary airway basal cells from non-COPD and COPD specimens and HBE-1 cell line were cultured as described in Supplemental Methods on collagen-coated tissue culture plates (unless otherwise indicated). All secretion assays were performed at 37°C and 5% CO_2_. Media were exchanged to fresh prewarmed BEGM at beginning of assay, and plates were incubated for 2 hours. Supernatant was clarified and cells were lysed in MPER (Pierce) supplemented with HALT protease inhibitor (Pierce). For all secretion assays, IL-33 protein was quantified in supernatant and lysate using R&D commercial ELISA assays (Supplemental Methods) with total assay protein (supernatant + lysate) and percentage secretion (supernatant/[supernatant + lysate] × 100) quantified based on standard curve. Some experiments were performed in polarized format using Transwell culture dishes (0.4 μm and 1.0 μm pore size). Calculations were performed similarly for polarized format, with total assay protein (apical + basal + lysate) and percentage secretion (apical or basal/[apical + basal + lysate] × 100).

For chemical inhibition assays, all chemicals were solubilized in DMSO and filter sterilized before use. As a control, DMSO was used at the highest concentration required for solubility in the assay. Inhibitors were preincubated with cells for 1 hour before beginning secretion assay and maintained in media during assay. Chemical concentrations used for inhibition assay were as follows: PBS; DMSO vehicle control (2.5%); BD GolgiPlug Brefeldin (1:1000), GolgiStop Monensin (1:1500), GW4869 (20 μM), Cambinol (10 μM), Spiroepoxide (5 μM), glutathione (5 μM), and 3-MA (5 μM) (manufacturer information and product number for chemicals are provided in Supplemental Table 2).

All secretion assays were performed with *n* = 5 biological replicates (unless otherwise indicated) and performed in triplicate.

### Alternaria mouse model.

WT 5-week-old male C57Bl/6 mice were purchased from The Jackson Laboratory. *Alternaria alternata* extract was purchased from Greer Laboratories and reconstituted and adjusted to 1 mg/ml solution in sterile saline (based on BCA assay, Pierce). GW4869 (N,N′-Bis[4-(4,5-dihydro-1H-imidazol-2-yl)phenyl]-3,3′-p-phenylene-bis-acrylamide dihydrochloride; MW 577.5 g/mol; MilliporeSigma) was reconstituted in DMSO (20 mg/mL stock) and diluted into sterile saline before use. Experiments included *n* = 11–14 mice per group and were repeated in triplicate. Five-week-old mice were either treated with 25 μg i.n. *Alternaria* extract (in 25 μl) or PBS control (25 μl) under isoflurane anesthesia every 48 hours for a total of 5 doses over 9 days. Mice in *Alternaria* groups were also treated with 100 μl of 0.5 mg/mL GW4869 in 2.5% DMSO/saline (50 μg/mouse; 2–2.5 μg/g body weight) or 100 μl of 2.5% DMSO/saline vehicle control every 24 hours for 9 days, beginning on the same day as the first dose of *Alternaria*. Mice were analyzed 24 hours after the final *Alternaria* dose, except for 1 hour after *Alternaria* BAL, for which mice were analyzed 1 hour after the fifth dose. No behavioral problems were observed during treatment. Mild body weight loss was observed in both *Alternaria* treatment groups (5%–10%), which was not different between groups and recovered by the end of the experiment. RNA extraction, qPCR and ELISA, and cell sorting are described in Supplemental Methods; tissue and cell preparations were lysed in TRIzol or TPER/HALT (Pierce) for analysis. BAL fluid was obtained by intratracheal instillation of 0.7 ml PBS with return volume of approximately 0.3–0.4 mL, and samples were centrifuged to pellet cells. Supernatants from *n* = 3 mice were analyzed by ELISA (mouse IL-33 DuoSet, R&D Systems) (normalized to total protein by BCA assay), and an equivalent volume of each replicate BAL supernatant was combined and concentrated with a 100 kDa concentrator and the pooled specimens were analyzed by TRPS and Western blot. Mouse *Alternaria* model experiments were performed with accompanying analysis in triplicate.

### Statistics.

For statistical analysis, 2-tailed Student’s *t* test was used for comparisons between 2 groups, and comparisons with 3 or more groups were analyzed using 1-way ANOVA. For all experiments, *P* < 0.05 was considered statistically significant. Correlation analysis was performed based on Pearson’s coefficient. For all data in which 3 or more independent measurements are reported, data are displayed as mean ± SEM.

### Study approval.

All human studies were conducted with protocols approved by the Washington University Institutional Review Board, and written informed consent was obtained from study participants. All experiments involving animals followed protocols approved by Washington University’s Institutional Animal Care and Use Committee.

## Author contributions

EKK, DFS, CEK, OAO, AB, and JAB designed and/or performed the experiments. EKK, DFS, CEK, OAO, CNS, and JAB prepared figures and wrote the manuscript. SLB and MJM contributed to preparation and editing of manuscript. DK, KLB, and DEB contributed to enrollment of human subjects and biobanking efforts. SLB, MJM, and MJH provided guidance with design/interpretation of experiments.

## Supplementary Material

Supplemental data

## Version 1 01/28/2021

In-Press Preview

## Version 2 02/22/2021

Electronic publication

## Version 3 02/23/2021

Updated Supplemental data
