# Supplementary material for: Epithelial IL-33 appropriates exosome trafficking for secretion in chronic airway disease
Source: JCI Insight. 2021 Feb 22;6(4):e136166. doi: 10.1172/jci.insight.136166 (PMC7934940; doi:10.1172/jci.insight.136166)
Supplement: Supplemental data [file jciinsight-6-136166-s253.pdf]

SUPPLEMENTARY MATERIALS

Supplementary Methods and References

Table S1. clinical characteristics of subjects without COPD (Non-COPD) and with severe (GOLD Stage IV) COPD, related to Figure 1.

Table S2. List of reagents and materials.

Figure S1. Live cell imaging and secretion assays for IL-33 variants in HBE-1 and primary airway basal cells, related to Figure 1.

Figure S2. Chemical inhibition, shRNA knockdown and CRISPR-based *SMPD3-/-* data and validation of assays, related to Figure 2.

Figure S3. Analysis of exosomes in COPD airway basal cell supernatants and COPD bronchial wash (BW) fluid, related to Figures 2, 3 and 6.

Figure S4. Expression of *IL33* isoforms in COPD, related to Figure 4.

Figure S5. In situ hybridization staining for $IL33^{\Delta 34}$ in COPD and non-COPD tissues with corresponding IL-33 protein immunostaining, related to Figure 4.

Figure S6. IL-33 immunostaining in COPD and non-COPD tissues, related to Figure 4.

Figure S7. NSMase2 and IL-33 immunostaining in lung tissue, related to Figure 5.

Figure S8. Analysis of mouse Alternaria airway disease model, related to Figure 7.

SUPPLEMENTARY METHODS

Quantitative PCR assays

To quantify alternate-spliced *IL33* transcripts in COPD specimens, we designed a series of quantitative PCR (qPCR) assays to probe the N-terminal truncation isoforms lacking exons 3 ($IL33^{\Delta 3}$), exon 4 ($IL33^{\Delta 4}$), exons 3-4 ($IL33^{\Delta 34}$) and exons 3-5 ($IL33^{\Delta 345}$). A specific assay could not be generated for exon 5 ($IL33^{\Delta 5}$) deleted isoform. Assay specificity was validated using individual plasmid standards (Figure S4A).

In general, RNA for qPCR was purified from lung homogenates and cell lysates using Trizol (Invitrogen) extraction and converted to cDNA template using a High-Capacity cDNA Archive® kit (Applied Biosystems), both according to manufacturer protocols. Target mRNA expression was quantified by qPCR assay fluorogenic probe-primer sets (labeled with 5'FAM and 3'-IowaBlack) as detailed in Table S2, either designed using the PrimerQuest online tool through Integrated DNA Technologies (IDT) website (*IL33* isoform-specific) or using IDT pre-designed, validated assays. QPCR was performed using the KAPA PCR Master Mix system (KAPA Biosystems). Samples were assayed with the 7500 Fast Real-Time PCR System and analyzed using Fast System Software (Applied Biosystems). Transcript copy/ml was quantified based on cycle threshold (Ct) for human *IL33* transcripts with reference plasmid standard, in cases where plasmid standard was not available, fold-change was calculated using the ΔΔCt method (relative to average ΔCt for control group). Samples that did not amplify with a Ct value threshold < 35 for a given target were not included in analyses. In all cases values were normalized to *GAPDH* (Applied Biosystems human and mouse GAPDH 20X assays).

In situ hybridization

A custom double-DIG labeled miRCURY LNA detection probe (Qiagen) was synthesized to target the $IL33^{\Delta 34}$ sequence (5'DIG-AGGTGAAATTCTTCCCAGCTTGAAA-3'DIG) at the exon2-exon 5 junction. The Qiagen miRCURY ISH detection system was chosen over conventional antisense RNA probe due to the increased sensitivity/stability of the probe and the short sequences allowed, thus minimizing hybridization to other IL-33 isoforms present in tissue. In situ hybridization was carried out according to manufacturer protocol using the miRCURY LNA miRNA ISH optimization kit (FFPE) 2 with U6 snRNA (positive control) and random sequence (negative control) LNA probes provided with the kit. Optimal signal-to-noise for

overexpressed *IL33*$^{\Delta 34}$ (relative to *IL33*^{full}) was obtained for the probe when used at 100 nM in the hybridization reaction (optimized conditions shown in Figure S5A). For the staining protocol, fresh cut FFPE slides were de-paraffinized in xylenes followed by proteinase K digestion (10 μg/ml) for 7 minutes. Probes were loaded in miRCURY hybridization buffer and incubated in a Dako hybridizer at 55°C for 1 hr. Serial SSC (saline sodium citrate) washes were performed at 55°C, followed by blocking with sheep serum and then incubation with alkaline phosphatase conjugated anti-DIG sheep FAB fragments (Roche) for 1 hr. Development using BCIP/NBT substrate supplemented with 20 mM levamisol was performed until optimal signal-to-noise was achieved, approximately 2 hrs. at 30°C. Slides were then counterstained with nuclear fast red and mounted for imaging.

Tissue and cell immunofluorescence

Tissues were fixed with 10% neutral buffered formalin, embedded in paraffin, cut into 5-µm sections, and adhered to charged slides. Sections were deparaffinized in Fisherbrand® CitroSolv®, rehydrated, and treated with heat-activated Vector Citrate antigen unmasking solution (Vector laboratories, Inc). After blocking (Tris buffered saline with 0.1% Triton X100 and animal free blocking solution, Vector labs), immunofluorescence staining was performed by incubating at 4° C overnight with primary antibodies at dilutions outlined in Table S2 followed by washing and incubating with secondary antibodies for 2 hr. at 25° C, including combinations of Alexa Fluor 488 nm, 594 nm or 647 fluorochromes (Invitrogen) or using Vector Laboratories fluorescent signal amplification kits (human IL-33 only). Immunohistochemistry staining followed a similar approach instead using alkaline phosphatase or horseradish peroxidase conjugated secondary antibodies (Vector Laboratories ImmPact staining kits) and Vector blue or AMEC red chromophores (Vector Laboratories), respectively. Cells on chamber slides were either imaged directly under live-cell conditions for cells transduced with mCherry-GFP labeled proteins or fixed for 5 min at room temperature with 4% paraformaldehyde and permeabilized in 0.1% Tris buffered saline, with detection using primary and secondary antibodies as above.

Live and fixed cell or tissue fluorescent imaging was performed on an Olympus IX83 inverted microscope with 40 and 60X objectives and a Hammamatsu Orca ER camera. Brightfield images were measured on an Olympus IX equipped with a Nikon color camera and 20X, 40X and 100X (oil) objectives.

Confocal imaging was performed using a Zeiss LSM 880 Confocal with Airyscan with 40X objective.

Cloning and fusion protein construct generation

For cloning of *IL33* isoforms from COPD tissue, PCR was performed using random-primer generated cDNA as template, and Pfu UltraII High Fidelity Master Mix (Agilent) according to manufacturer protocol. PCR products were separated on 1% agarose gel and visualized with ethidium bromide. Discernable bands amplified with IL-33 coding sequence primers (exon 2 and 8 with NdeI and XhoI restriction sites, Table S2) were extracted using Qiagen gel extraction kit, digested with corresponding restriction enzymes, ligated into pET23b expression vector and transformed into NEB 10-beta *E. coli* for plasmid isolation (New England Biolabs). Fifteen individual clones were isolated by miniprep (Qiagen) and submitted for Sanger sequencing, yielding duplicate clones of $IL33^{full}$, $IL33^{\Delta 34}$ and $IL33^{\Delta 345}$. Isoforms not cloned from COPD cells were generated by PCR by engineering a SpeI restriction site into the $IL33^{\Delta 4}$ exon 3-5 boundary followed by quick-change mutagenesis (Stratagene QuickChange Lightning) or using an internal BamHI site at the exon 2-4 boundaries of $IL33^{\Delta 3}$ and AgeI at the exon 4-6 boundaries of $IL33^{\Delta 5}$. The non-natural $IL33^{\Delta 2}$ construct was generated using cloning primers to exclude the second exon segment. These products were used as templates for cloning of fusion proteins and as qPCR plasmid standards.

Lentiviral constructs were designed to express IL-33 proteins as dual tagged fusions with N-terminal mCherry and C-terminal eGFP (eGFP monomeric variant) or N-terminal Flag (DYKDDDDK) and C-terminal 6His (6XHis) tags. For fluorescent fusion proteins, mCherry, IL-33 variants and GFP (Clontech mCherry and monomeric eGFP) were amplified with complimentary primers and Gly-Gly-Gly-Ser (GGGS) linkers as follows: NdeI-mCherry-(GGGS)-EcoRI-IL-33-KpnI-(GGGS)-GFP-NotI. PCR products were digested, ligated into the lentiviral vector(s) pCDH-EF1-MCS-IRES-Puro/Neo (System Biosciences) and transformed into NEB stable cells (New England Biolabs). An analogous approach was taken for Flag/His dual-tagged constructs (without GGGS linkers). Constructs were generated using the pCDH-EF1-MCS-IRES-Puro vector in all cases except where double selection was required for use with shRNA lentiviruses, in which case, constructs were cloned into pCDH-EF1-MCS-IRES-Neo.

Recombinant IL-33 protein expression

We validated the anti-IL-33 NTD antibody targeting exons 3-4 (Abcam) and anti-CTD antibody targeting exons 5-8 (R&D systems) by western blot using HEK293T lysates overexpressing *IL33* isoforms $IL33^{full}$ (calculated molecular weight (MW) 34 kilodaltons (kDa)) and $IL33^{\Delta 34}$ (calculated MW 25 kDa) compared to commercially available CTD product (amino acids 110-270, 17 kDa, Peprotech). Proteins were transiently expressed in HEK293T cells by combining a 2:1 (µl reagent:µg plasmid) ratio of plasmid DNA with 293Fectin (Life Technologies) in Optimem (Gibco) according to manufacturer protocol and transfection complexes were added to cells at 70% confluence. Cells lysates were used in analysis 24 hours later. The CTD-targeting antibody reacted with all three forms, while the NTD antibody reacted only with the full-length protein, thus validating these reagents to probe endogenous IL-33 fragments.

Recombinant IL-$33^{\Delta 34}$ for exosome binding experiments was cloned into pET21a (Novagen) with an N-terminal combined 6-His and AviTag™ sequence (HHHHHHGLNDIFEAQKIEWHE) and $(GGS)^2$ linker. The IL-$33^{\Delta 34}$ construct was transformed into Rosetta2(DE3) *E. coli*, (Novagen) under antibiotic selection and 2L of cells were grown in Luria Broth (LB) at 37°C to an OD_{600} of ~0.8 before induction with 0.5mM of IPTG per manufacturer protocols. Cells were shaken at 16°C overnight, then pelleted and resuspended in 50 mM Na_2HPO_4 pH 8.0, 300mM NaCl, 5mM imidazole, 10mM β-ME and 10% glycerol. Lysozyme, DNAse, $MgCl_2$, 1mM PMSF and 0.1% Triton X-100 were added prior to sonication. Following centrifugation at 12,000 x g, supernatants were passed over NiNTA superflow resin (Qiagen). Resin was washed with 50 mM Na_2HPO_4 pH 8.0, 300 mM NaCl, 40 mM imidazole, 10 mM β-ME and 10% glycerol, and bound protein eluted in 50 mM Na_2HPO_4 pH 8.0, 300 mM NaCl, 300 mM imidazole, 10 mM β-ME and 10% glycerol supplemented with protease inhibitor cocktail (Sigma). IL-$33^{\Delta 34}$-birA-6His was further purified by size exclusion chromatography (SEC) using a Superdex™ 75 Increase column. Purified IL-$33^{\Delta 34}$ was exchanged into 100mM Tris-HCl pH 7.5, 200mM NaCl, 5mM $MgCl_2$, 10mM β-ME and 10% glycerol for subsequent birA enzymatic biotinylation as previously described (1).

IL-33 ELISA

Lung tissues were homogenized in TPER (Pierce) with HALT protease inhibitors (Pierce) and cultured cells were lysed in MPER (Pierce) with HALT; samples were centrifuged at >10,000x*g* for 5 min to clarify and

further diluted as necessary based on dynamic range of assay. Human IL-33 ELISA was performed using IL-33 DuoSet® (R&D Systems) according to the manufacturer's protocol. For endogenous IL-33 and all variants harboring the exon 5 segment, the monoclonal DuoSet® assay was used. For variants lacking exon 5, the IL-33 polyclonal DuoSet® was required for detection (due to monoclonal epitope residing within exon 5), and throughout figures this is indicated with angled stripe on data bars. Chromogenic signal was developed using TMB substrate (SeraCare) and absorbance at 450 nm measured on a BioTek Synergy H1 system. Where indicated, IL-33 protein level was normalized to total protein using BCA assay (Pierce).

NSMase2 activity assay

NSMase2 activity was measured by colorimetric assay using a commercial kit (Abcam). Tissue and cell lysates were added to 96-well assay plates along with substrate and buffers provided in the kit and assay performed per manufacturer protocol. Bronchial wash samples were also tested but did not demonstrate signal above background (data not shown). Plates were incubated for recommended time frame and read on a BioTek Synergy H1 system at 695 nm. NSMase2 activity was calculated based on standard curve provided in kit.

Western blot

For analysis of truncated IL-33 proteins in biological samples, equivalent amounts of cell and tissue lysate or bronchial wash fluid were prepared for western blot and ELISA for each sample. Gel electrophoresis was performed using NuPAGE™ 4-12% Bis-Tris Protein Gels (Invitrogen), followed by transfer to nitrocellulose membrane using iBlot™ 2 Transfer Stacks (Invitrogen) and an iBlot™ 2 Dry Blotting System (Invitrogen). Membranes were washed in PBS 0.1% Tween-20 (PBST) and stained in PBST with 2% BSA in the SNAP I.D.® 2.0 Protein Detection System (Millipore). Antibodies were used at dilutions specified in Table S2 with overnight incubation at 4°C. Membranes were washed and incubated with 1:5000 dilution of appropriate HRP-conjugated secondary antibodies (Santa Cruz) at room temperature. Western blots were developed using ECL Substrate (Pierce) and chemiluminescence detected by exposure to autoradiography film.

Airway epithelial cell culture

Primary-culture airway basal cells were established from large airway (1st-3rd generation) tracheobronchial specimens as described (2, 3) and cultured in 2D submerged format either on collagen-coated plastic or Transwell (Corning 0.4 μm, Millipore 1.0 μm) inserts. Airway basal cells were maintained at 37°C 5% CO2 in BEGM medium without retinoic acid (RA) or serum (4). HBE cell lines were grown in LHC-8 medium (Gibco). Cells were seeded at 20,000 cells/well and grown to >90% confluence on plastic or 100% confluence in Transwells prior to study. Airway cells were lysed either in Trizol or M-PER/HALT for mRNA and protein analysis.

Generation of lentiviruses and transduction of cells

Lentiviruses were generated for fusion protein expression in primary airway basal cells and generation of stable HBE-1 lines using the LentiSuite system (Systems Biosciences) according to manufacturer protocol. Briefly, HEK293Ts were transfected with transfer plasmid pPACKH1 using PureFection reagent. Virus was collected at 48-72 hours post transfection, clarified by centrifugation at 3000xg and virus precipitated with PEG-it solution. Titer of the virus was measured using Lenti Go-Stix and aliquots stored at -80°C until use. Airway epithelial cells (HBE-1 or primary airway basal cells) cultured on collagen coated tissue culture plates were transduced according to published protocols (5). Briefly, they were trypsinized, centrifuged and resuspended in BEGM media supplemented with 1 μg/ml protamine. Lentivirus was added to cells resuspended at $1x10^6$ cells/ml at MOI 1-10 and cells were returned to collagen-coated plates for 24 hrs. Media and virus was then removed and fresh media containing the appropriate antibiotic selection (puromycin at 0.5 μg/ml or G418 at 250 μg/ml) and 10 μM Y27632 (primary cells only, Stem Cell Technologies) was added. Transfection efficiency ranged from 10-50% and cells were allowed to completely recover to 80-90% confluence and protein expression verified prior to use. For shRNA assays, low passage primary human airway basal cells were trypsinized and plated with high-titer shRNA lentiviruses purchased from Santa Cruz and protamine at 1 μg/ml. After 24 hours, virus was removed and media replaced with BEGM containing puromycin at 0.5 μg/ml and 10 μM Y-27632. Media was changed daily until cells had visibly recovered and were sufficient in number to undergo a repeated round of infection. The process was repeated with Flag-IL-$33^{\Delta34}$-His lentivirus. Cells underwent a second round of selection

with G418, allowed to recover, and then were plated on 96-well plates for ELISA assay or chamber slides for imaging.

Generation of stable mCherry-IL-33-GFP expressing HBE-1 cell lines

We initially chose the immortalized human airway epithelial line HBE-1 (6) to develop these assays, as we determined that this line exhibited in vitro behavior and markers that best approximated primary airway basal cells (compared to A549 and BEAS2B) and could be grown in serum-free medium. Stable mCherry-IL-33-GFP HBE-1 lines were developed to establish a robust system to probe IL-33 cellular trafficking under stable expression conditions. Cells were transduced with lentiviruses and passaged under appropriate antibiotic selection for 2-3 weeks, then sorted selecting for the top 0.5-1% of double-positive cells on a MoFlo (DAKO-Cytomation) sorter. Cells were recovered and expanded and post-sort cells exhibited 100% expression of fusion proteins, which was stable without selection for greater than 10 passages.

CRISPR generation of *SMPD3-/-* HBE-1 cell line

SMPD3$^{-/-}$ HBE-1 cells were generated using CRISPR-Cas9 ribonucleoprotein (RNP) targeting exon 1 of *SMPD3* with guide RNA (Alt-R sgRNA, Integrated DNA technologies) and Cas9 protein (QB3 Berkeley MacroLab, UC Berkeley). Transfection was performed on the Lonza Nucleofector 4D X Unit using 250K cells in a 20 μl cuvette with solution P3 and program CA-137. Targeted deep-sequencing was performed to validate the KO pool and clones as previously described(7). Initial pools demonstrated 76% NHEJ with 25% wild-type alleles present. Flag-IL-33$^{\Delta34}$-His secretion was then used as functional readout to enrich for *SMPD3* deletion by limiting dilution assay. After 4 rounds of limiting dilution, subsets with the lowest level of secretion were genotyped and verified to have 0% residual wild-type expression with a combination of 3 indels present (Figure S2G) and were subsequently used for secretion assay.

HMC1.2 assays

The human mast cell line HMC1.2 (8) was purchased from Millipore and cultured in IMDM media supplemented with penicillin-streptomycin, 10% fetal calf serum and α-thioglycerol according to manufacturer protocol. For Flag-IL-33$^{\Delta34}$-His secretion, lentiviral transduction and selection were performed

as above for airway epithelial cells. Cells were plated at $3x10^5$ cells per well in 96-well assay plates, and assays were performed for 2 hrs. at 37°C 5% CO2 with a minimum of 3 biological replicates, repeated in triplicate. For IL-33-induced stimulation, concentrated BW-purified protein from Superose 6 column and concentrator flow-through (negative control) and commercial IL-33 CTD (Peprotech) were diluted into HMC media and re-quantified prior to assay (to ensure matched concentrations due to low quantity of purified endogenous BW IL-33 protein). Cells were incubated overnight and supernatants harvested for IL-8 quantification by ELISA (R&D Systems). Under conditions where IL-1RL1 blockade was performed, blocking antibody was added at 100 ng/ml to HMC cells 2 hr. prior to performing assay and was maintained in the culture conditions during assay.

Mouse lung cell sorting and FACS analysis

Lungs were prepared for FACS analysis as previously described (9). Briefly, single-cell suspensions were generated from minced lung tissue subjected to collagenase (Liberase Blendzyme III, Roche), hyaluronidase (Sigma Aldrich), and DNAse I (grade II, Roche) with digestion for 45 minutes at 37°C. After FcR blockade, lung cell suspensions were incubated with labeled antibodies and sorted using a Moflo sorter. Mouse cells were immunostained with AF700-conjugated anti-mouse CD45 (Biolegend), APC-conjugated anti-mouse lineage cocktail (BD Biosciences), BV421-conjugated anti-Thy1.2 (Biolegend) and PE-conjugated anti-mouse IL1RL1/ST2 (Biolegend). Flow cytometry results were analyzed using FlowJo software (TreeStar).

SUPPLEMENTARY REFERENCES

1. Kober DL, Alexander-Brett JM, Karch CM, Cruchaga C, Colonna M, Holtzman MJ, et al. Neurodegenerative disease mutations in TREM2 reveal a functional surface and distinct loss-of-function mechanisms. *Elife.* 2016;5.
2. You Y, Richer EJ, Huang T, and Brody SL. Growth and differentiation of mouse tracheal epithelial cells: selection of a proliferative population. *Am J Physiol Lung Cell Mol Physiol.* 2002;283(6):L1315-21.
3. Rock JR, Onaitis MW, Rawlins EL, Lu Y, Clark CP, Xue Y, et al. Basal cells as stem cells of the mouse trachea and human airway epithelium. *Proc Natl Acad Sci U S A.* 2009;106(31):12771-5.
4. Randell SH, Walstad L, Schwab UE, Grubb BR, and Yankaskas JR. Isolation and culture of airway epithelial cells from chronically infected human lungs. *In Vitro Cell Dev Biol Anim.* 2001;37(8):480-9.

5. Baumlin-Schmid N, Salathe M, and Fregien NL. Optimal Lentivirus Production and Cell Culture Conditions Necessary to Successfully Transduce Primary Human Bronchial Epithelial Cells. *J Vis Exp.* 2016(113).
6. Yankaskas JR, Haizlip JE, Conrad M, Koval D, Lazarowski E, Paradiso AM, et al. Papilloma virus immortalized tracheal epithelial cells retain a well-differentiated phenotype. *Am J Physiol.* 1993;264(5 Pt 1):C1219-30.
7. Sentmanat MF, Peters ST, Florian CP, Connelly JP, and Pruett-Miller SM. A Survey of Validation Strategies for CRISPR-Cas9 Editing. *Sci Rep.* 2018;8(1):888.
8. Sundstrom M, Vliagoftis H, Karlberg P, Butterfield JH, Nilsson K, Metcalfe DD, et al. Functional and phenotypic studies of two variants of a human mast cell line with a distinct set of mutations in the c-kit proto-oncogene. *Immunology.* 2003;108(1):89-97.
9. Byers DE, Alexander-Brett J, Patel AC, Agapov E, Dang-Vu G, Jin X, et al. Long-term IL-33-producing epithelial progenitor cells in chronic obstructive lung disease. *J Clin Invest.* 2013;123(9):3967-82.

Table S1. Clinical characteristics of lung transplant donors without COPD (Non-COPD) and recipients with severe (GOLD Stage IV) COPD.

Characteristics	Non-COPD	COPD
Number per group	13	27
Mean age (range)	39.13 (16-68)	61.43 (51-75)
Male:Female	8:5	12:15
FVC (L)	–	2.12 (0.83)
FEV1 (L)	–	0.52 (0.17)
FEV_1/FVC (%)	–	22.74
Pack-years (range)	2.53 (0-20)	7.68 (0.67-34)

Table S2: List of reagents and materials.

Reagent	Supplier	Reference/Identifier
**Antibodies**		
Human IL-33 rabbit polyclonal (1:50 IF)	Sigma Aldrich	HPA024426
Human IL-33 goat monoclonal (1:1000 WB)	R&D systems	MAB36253
Human IL-1RL1/ST2 mouse monoclonal (B)	Proteintech	60112-1
Human nSMase2 mouse monoclonal (1:50 IF)	Santa Cruz	sc-166637
Human VPS4A (A-11) mouse (1:100 IF)	Santa Cruz	Sc-393428
Human LAMP2 (ABL-93) mouse (1:100 IF)	Santa Cruz	Sc-20004
Human CD9 (H19a) mouse (1:1000 WB)	Biolegend	312102
Human CD9 (MM2/57) (1:100 IF)	Millipore	CBL162
Anti-DYKDDDDK-AF594	Biolegend	637313
Human EpCAM/CD326 (1:1000 WB)	Biolegend	324201
Anti-Mouse lineage cocktail (1:75 FC)	BD	558074
Human IL-1RL1 monoclonal (1:75 FC)	Biolegend	145303
Human Thy1.2/CD90 monoclonal (1:75 FC)	Biolegend	105341
Mouse IL-33 goat polyclonal (1:50 IF)	R&D systems	AF3625
IF = immunofluorescence, WB = western blot, FC = flow cytometry B = blocking		
**Commercial lentiviruses**		
Scrambled shRNA	Santa Cruz	sc-108080
nSMase2 shRNA	Santa Cruz	sc-62655-V
VPS4 shRNA	Santa Cruz	sc-106731-V
LAMP2 shRNA	Santa Cruz	sc-29390-V
**Biological Samples**		
Primary human basal cells	BJH	N/A
Control and COPD lung tissue	BJH, UNMC	N/A
Control and COPD Bronchial wash fluid	BJH	N/A
**Chemicals, Peptides, and Recombinant Proteins**		
IL-33	Peprotech	200-33
GW4869	Sigma Aldrich	D1692
Brefeldin A	BD Biosciences	51-2301KZ
Monensin	BD Biosciences	51-2092KZ
4-methylumbelliferone	Sigma Aldrich	M1381
Cambinol	Sigma Aldrich	C0494
Spiroepoxide	Santa Cruz	sc-202721
Glutathione	Sigma Aldrich	1294820
3-methyladenine	Sigma Aldrich	M9281
Alternaria alternata extract	Greer laboratories	XPM1D3A2.5
**Commercial Assays**		
R&D IL-33 ELISA duoset	R&D Systems	DY3625B (human)<br>DY3626 (mouse)
R&D IL-8 ELISA duoset	R&D Systems	DY208 (human)

**Cell Lines**		
HBE-1	Yankaskas et al, 1993	Gift from MJ Holtzman lab
HMC 1.2	Millipore	SCC062
**Oligonucleotides**		
**Cloning primers**		
5'-EcoRI-Kozak-FLAG-hIL33	GGAATTCCGCCACCATGGATTACAAGGATGACGATGACAAGCCT AAAATGAAGTATTCA	
3’-hIL33-6His-stop-Not1	ATAGTTTAGCGGCCGCTCAATGGTGATGGTGATGATGAGTTTCA GAGAGCTTAAACAAG	
5’-NheI-Kozak-mCherry	CTAGCTAGCGCCACCATGGTGAGCAAGGGCG	
3’-EcoRI-mCherry	GGAATTCCTTGTACAGCTCGTCC	
5'-EcoRI-hIL33-GS-link	GGAATTCGGTGGTGGCGGTTCAAAGCCTAAAATGAAGTATTCA	
3'-hIL33-Kpn	GGGGTACCAGTTTCAGAGAGCTTAAACAA	
5’-Kpn-GSS-linker-GFP	GGGGTACCGGTGGTGGCGGTTCAGTGAGCAAGGGCGCCGAGG	
3’-GFP-stop-NotI	ATAGTTTAGCGGCCGCTCACTTGTACAGCTCATCCATGC	
hIL-33 Δ Exon4 3’-segment1	ACTAGTACCTGTTTTCAGTGAAGGCCTTTTG	
hIL-33 Δ Exon4 5’-segment2	ACTAGTCCTATTACAGAGTATCTTGCTTCTCTAAGCAC	
hIL-33 Δ Exon3 3’-segment1	CGGGATCCCAGCTTGAAACACAAGGCTTTGC	
hIL-33 Δ Exon3 5’ segment2	CGGGATCCAGAAAGCACAAAAGACATCTGG	
5'-EcoRI-Kozak-FLAG-hIL33 Δ Exon2	GGAATTCCGCCACCATGGATTACAAGGATGACGATGACAAATCC CAACAGAAGGCCAAAGAAG	
**Human *SMPD3* CRISPR targeting and genotyping primers**		
SMPD3 sgRNA	GCCCTACATCTATTCACGGC	
XCB616e.F	TTCCATGCTACTGGCTGGTG	
XCB616e.R	CCGCGCTTGGGTGTTAAAAA	
**LNA probe (ISH)**		
IL33 Δ Exon 34	5’DIG-AGGTGAAATTCTTCCCAGCTTGAAA-3’DIG	
**qPCR assays**		
Lab designed assays		
Human *IL33* full		
Forward Primer	CCCAACAGAAGGCCAAAGAA	
Probe	TTTATGAAGCTCCGCTCTGGCCTT	
Reverse Primer	GGTGGTTTCTCTCCTAAAGTAACAG	
IL33 Δ Exon 4		
Forward Primer	ACAGGTATTAGTCCTATTACAGAGTAT	
Probe	CTTGCTTCTCTAAGCACATACAATGATCA	
Reverse Primer	TCATAACTTTCATCCTCCAAAGC	
IL33 Δ Exon 3		
Forward Primer	TCAAGCTGGGATCCAGAAAG	
Probe	ATCTGGTACTCGCTGCCTGTCAAC	

Reverse Primer	GATATACCAAAGGCAAAGCACTC	
IL33 Δ Exon 34		
Forward Primer	CACAGCAAAGTGGAAGAACAC	
Probe	AGCTTGAAACACAAGGCTTTGCTTGC	
Reverse Primer	TACTCTGTAATAGGTGAAATTCTTCCC	
IL33 Δ Exon 345		
Forward Primer	GTGTTTCAAGCTGGGAAATAAGG	
Probe	TGAATCAGGTGACGGTGTTGATGGT	
Reverse Primer	GGACTCAGGGTTACCATTAACA	
SMPD3		
Forward Primer	GACGTGGCCTATCACTGTTAC	
Probe	TGTTTCTCAAGGTGCAGGTGGGAA	
Reverse Primer	CGATGTACCCGACGATTCTTT	
LAMP2		
Forward Primer	GATACTTGTCTGCTGGCTACC	
Probe	TCCTGAGTGATGTTCAGCTGCAGC	
Reverse Primer	GCCTGTGGAGTGAGTTGTATT	
VPS4A		
Forward Primer	TGAAGGTGGGAGGTTGATTG	
Probe	ATGTCAACAGCCAGACAGGGCT	
Reverse Primer	CAGCAGAGGGAACTCTGTATTG	
Mouse *Il33*		
Forward Primer	CAGCTGCAGAAGGGAGAAAT	
Probe	CACGGCAGAATCATCGAGAAACCTGA	
Reverse Primer	GGGAAATCTTGGAGTTGGAATACT	
IDT PrimeTime® predesigned qPCR assays		
Muc5ac	Mm.PT.58.42279692	
Il13	Mm.PT.58.31366752	
Tslp	Mm.PT.58.41321689	
Il5	Mm.PT.58.41498972	
Smpd3	Mm.PT.58.11603057	
**Constructs**		
human IL-33 Full Length	pCDH - puro	Cterm-GFP
human IL-33 Full Length	pCDH - puro	Cterm-GFP, N-term-mCherry
human IL-33 Full Length	pCDH - puro	Nterm-flag, Cterm-6his
human IL-33 Δ Exon3-4	pCDH - puro	Nterm-mCherry, Cterm-GFP
human IL-33 Δ Exon3-4	pCDH - puro	Nterm-flag, Cterm-6his
human IL-33 Δ Exon3-4	pCDH - neo	Nterm-flag, Cterm-6his
human IL-33 Δ Exon3-4	pCDH - neo	Nterm-mCherry, Cterm-GFP
human IL-33 Δ Exon2	pCDH - puro	Nterm-flag, Cterm-6his
human IL-33 Δ Exon2	pCDH - puro	Nterm-mCherry, Cterm-GFP
human IL-33 Δ Exon3	pCDH - puro	Nterm-flag, Cterm-6his
human IL-33 Δ Exon3	pCDH - puro	Nterm-mCherry, Cterm-GFP
human IL-33 Δ Exon4	pCDH - puro	Nterm-flag, Cterm-6his
human IL-33 Δ Exon4	pCDH - puro	Nterm-mCherry, Cterm-GFP

human IL-33 Δ Exon5	pCDH - puro	Nterm-flag, Cterm-6his
human IL-33 Δ Exon5	pCDH - puro	Nterm-mCherry, Cterm-GFP
human IL-33 Δ Exon3-5	pCDH - puro	Nterm-flag, Cterm-6his
human IL-33 Δ Exon3-5	pCDH - puro	Nterm-mCherry, Cterm-GFP
human IL-33 Δ Exon3-4	pET21	N-term-birA-6His
**Other**		
Sphingomyelinase Assay Kit (Colorimetric)	Abcam	ab138876

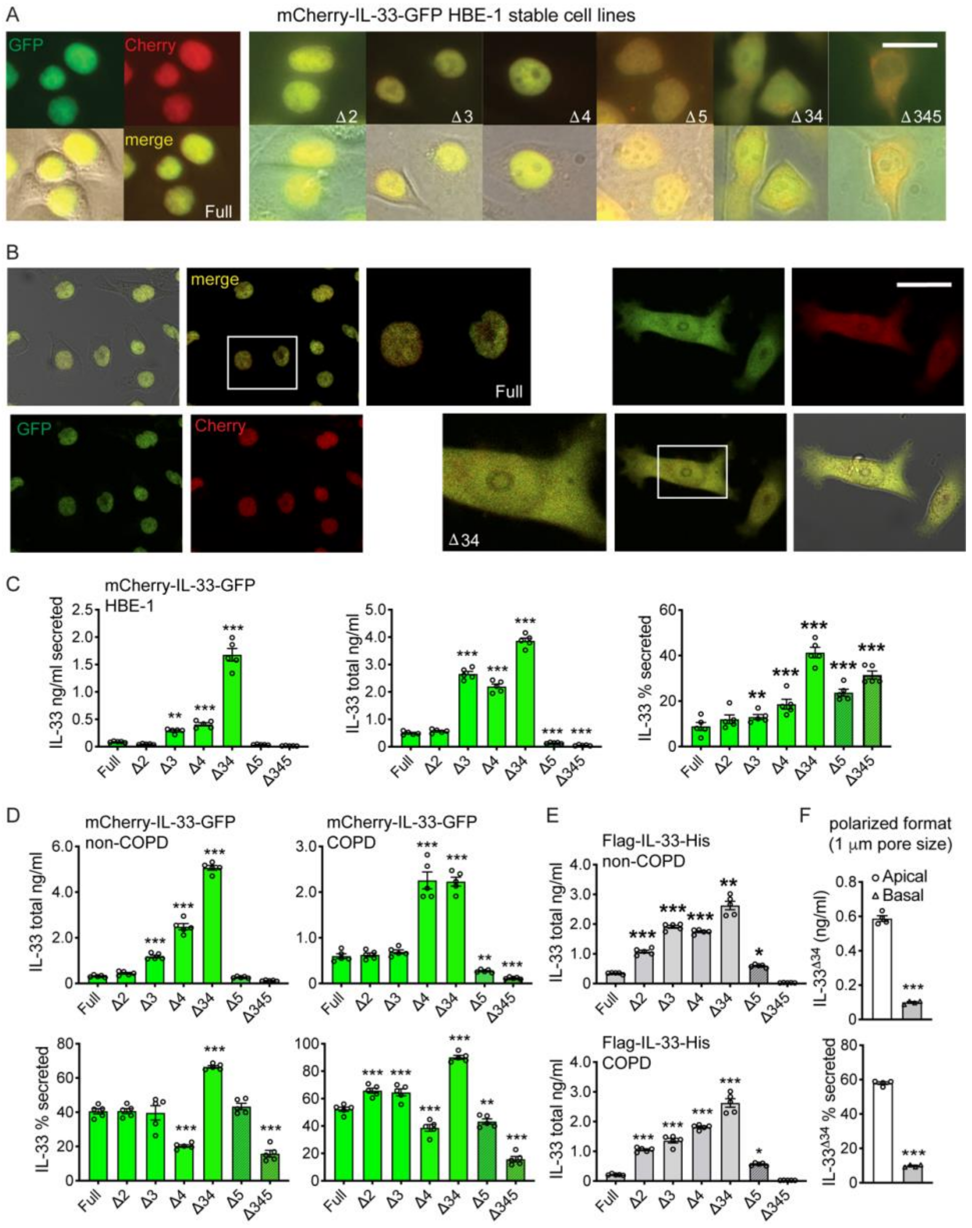

Figure S1. Secretion of IL-33 variants in HBE-1 and COPD airway basal cells, related to Figure 1. A) Live cell imaging of stable HBE-1 mCherry-IL33-GFP variants with phase contrast overlay for single exon deleted variants: IL-33^{full}, IL-33$^{\Delta3}$, IL-33$^{\Delta4}$, and IL-33$^{\Delta5}$ (and a non-natural IL-33$^{\Delta2}$ deletion variant) and compound deleted variants IL-33$^{\Delta34}$ and IL-33$^{\Delta345}$. Individual GFP and mCherry signal, as well as merged yellow signal shown for IL-33^{full} demonstrating overlapping signal for dual tags. Nuclear localization of IL-33^{Full}, IL-33$^{\Delta2}$, IL-33$^{\Delta3}$, IL-33$^{\Delta4}$ and IL-33$^{\Delta5}$ fusion proteins is observed, while mixed nuclear and cytoplasmic distribution is seen for IL-33$^{\Delta34}$ and IL-33$^{\Delta345}$ as in Figure 1C. Scale bar: 10 μm. **B**) Confocal imaging of stable HBE-1 mCherry-IL-33^{Full}-GFP and mCherry-IL-33$^{\Delta34}$-GFP variants with same cellular distribution as observed for epifluorescent imaging. **C**) ELISA secretion assay for stable mCherry-IL33-GFP HBE-1 cell lines, similar to primary cells in Figure 1D (n=5). IL-33 protein was measured using monoclonal (R&D Systems) and polyclonal (R&D Systems, angled stripe bars) assays. Absolute (ng/ml) and percentage secreted were most abundant for IL-33$^{\Delta34}$ variant, which also exhibited highest protein levels. **D**) and **E**) Total protein and percentage secretion for mCherry-IL33-GFP and Flag-IL-33-His variants in non-COPD and COPD cells, corresponding to Figure 1D&E, with IL-33$^{\Delta34}$ variant demonstrating most abundant absolute and percentage secretion (n=5). **F**) Secretion assay for non-COPD cells expressing Flag-IL-33$^{\Delta34}$-

His protein using Falcon® cell culture inserts with 1 μm pore size. Protein is detected in both apical and basal fractions with an apical predominance as in Figure 1G, shown as absolute (ng/ml) and percentage secreted protein (n=4). Data points displayed with mean ± SEM. Statistical analysis: one-way ANOVA (C, D, E), t-test (F), *P*-values: *$P < 0.05$, **$P < 0.01$, ***$P < 0.001$.

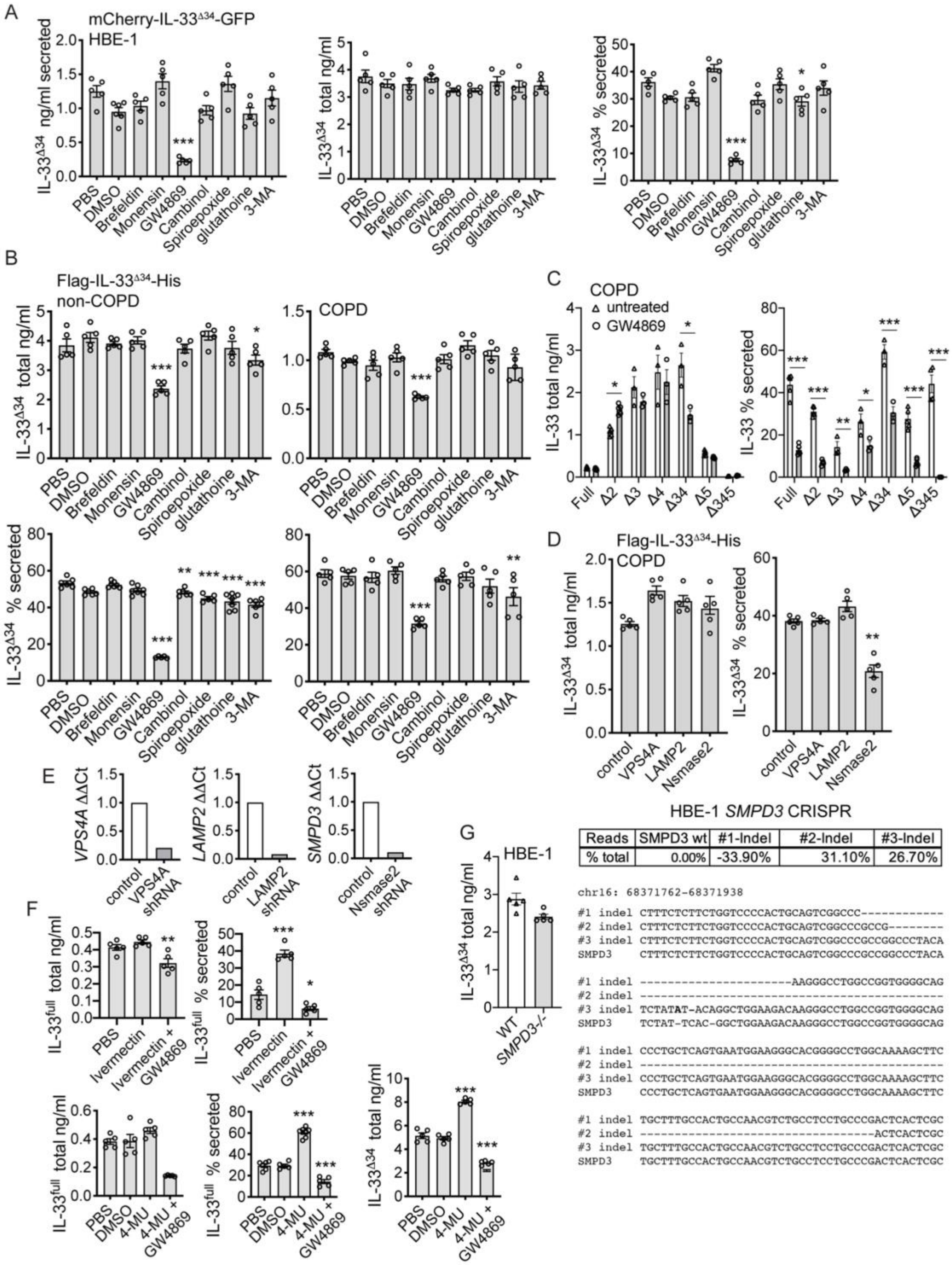

Figure S2. Inhibition of nSMase2 with validation of assays, related to Figure 2. **A**) Chemical inhibition of mCherry-IL-33$^{\Delta34}$-GFP secretion in HBE-1 stable cells treated with PBS, vehicle control (DMSO), Golgi transport inhibitors (Brefeldin, Monensin), nSMase inhibitors (GW4869, Cambinol, Spiroepoxide), glutathione, and autophagy inhibitor (3-MA) as in Fig 2A (concentrations reported in Methods). ELISA-quantified secreted protein, total and percentage secreted demonstrate marked inhibition by GW4869, similar to Figure 2A (n=5). **B**) Total protein levels and percentage secretion for COPD and non-COPD cells treated with inhibitors corresponding to Figure 2A (n=5). A decrease in total assay protein with GW4869 treatment is noted, but percentage secretion (adjusted for total protein) remains significant. **C**) Secretion assay for COPD cells treated with GW4869 and control (DMSO), demonstrating stable total protein and significant decrease in percentage secretion for variants, corresponding to Figure 2B (n=3-5). **D**) Flag-IL-33$^{\Delta34}$-His secretion assay in COPD cells with shRNA knockdown demonstrating stable total protein level and decreased percentage secretion with nSMase2 shRNA, corresponding to Figure 2D (n=5). **E**) Validation of shRNA-mediated knockdown of targets in COPD cells by qPCR. **F**) Secretion assay for Flag-IL-33^{full}-His and Flag-IL-33$^{\Delta34}$-His in COPD cells corresponding to Figure 3A-D, demonstrating modest effect of Ivermectin and 4-MU on total protein levels, with modest decrease in total protein with GW4869 treatment, yet significant blockade based on percentage secretion (n=5). **G**) Total Flag-IL-33$^{\Delta34}$-His protein in HBE wild-type and *SMPD3-/-* secretion assay (n=5) and percentage of total reads for indels #1-3 in *SMPD3-/-* line that exhibit large deletions and insertions, all expected to result in non-functional protein product. Data points displayed with mean ± SEM. Statistical analysis: one-way ANOVA (A, B, D, F), t-test (C, G), *P*-values: $^{*}P < 0.05$, $^{**}P < 0.01$, $^{***}P < 0.001$.

A

TRPS analysis (fraction 7)	293T cells	COPD 3 bronchial wash	COPD cells control shRNA	VPS4A shRNA	LAMP2 shRNA	nSMase2 shRNA
Mean (St. Dev.)	150(41)	136(25)	125(13)	153(21)	143(31)	-
Mode	124	127	122	142	132	-
Maximum	515	279	154	276	259	-
Minimum	105	85	99	107	106	-
Concentration (particles/ml x 10^9)	2.50	1.70	0.53	9.3	0.65	#

B

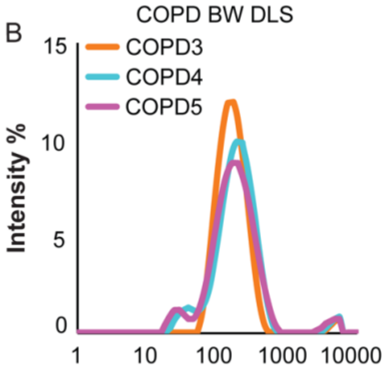

C

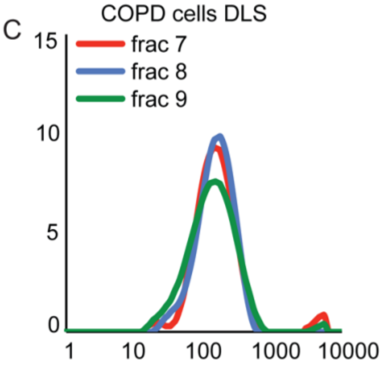

D

DLS Analysis (fraction 7)	Z-average nm (maximum intensity peaks)	PdI
COPD 3 bronchial wash	138.5 (164)	0.23
COPD 4 bronchial wash	144.2 (190)	0.39
COPD 5 bronchial wash	127.9 (141)	0.27
COPD cells	127.9 (141)	0.27
control shRNA	100.2 (105)	0.12
VPS4A shRNA	198.4 (141, 955)	0.68
LAMP2 shRNA	155.8 (11, 220)	0.73
NSmase2 shRNA	46 (13, 342)	1

Figure S3. Analysis of exosomes related to Figures 2, 3 and 6. Tunable Resistive Pulse Sensing (Izon qNano) and Dynamic Light Scattering (DLS, Malvern NanoS) were used to analyze concentration and size distribution of exosomes from COPD bronchial wash (BW) fluid and airway basal cell culture supernatants. **A**) TRPS analysis of particles < 150 nm size range are tabulated for COPD BW and airway basal cell supernatants under baseline and shRNA knockdown conditions, shown for fraction 7, the typical peak elution of exosomes from the column. Representative histograms are shown in Figure 2F and 6D. HEK293T derived particles are shown for comparison. Secreted exosome quantity was reduced to #undetectable level (<10^7 particles/ml) under nSMase2 shRNA conditions. Dynamic light scattering particle size histograms over the range of 10^1 to 10^4 nm (Malvern NanoS) are shown for comparison to TRPS method for **B**) COPD BW (n=3 specimens) and **C**) COPD basal cell supernatants (fractions 7-9). Results are tabulated in **D**) for COPD BW specimens and COPD basal cell supernatants under baseline and shRNA knockdown conditions. Results obtained using TRPS and DLS are consistent with respect to vesicle size distribution. TRPS offers measurement of vesicle concentration with more limited size resolution, while DLS is not dependent on filter cutoffs and samples a broader dynamic range, demonstrating increase in vesicle size (maximum intensity peaks) and heterogeneity under shRNA knockdown conditions.

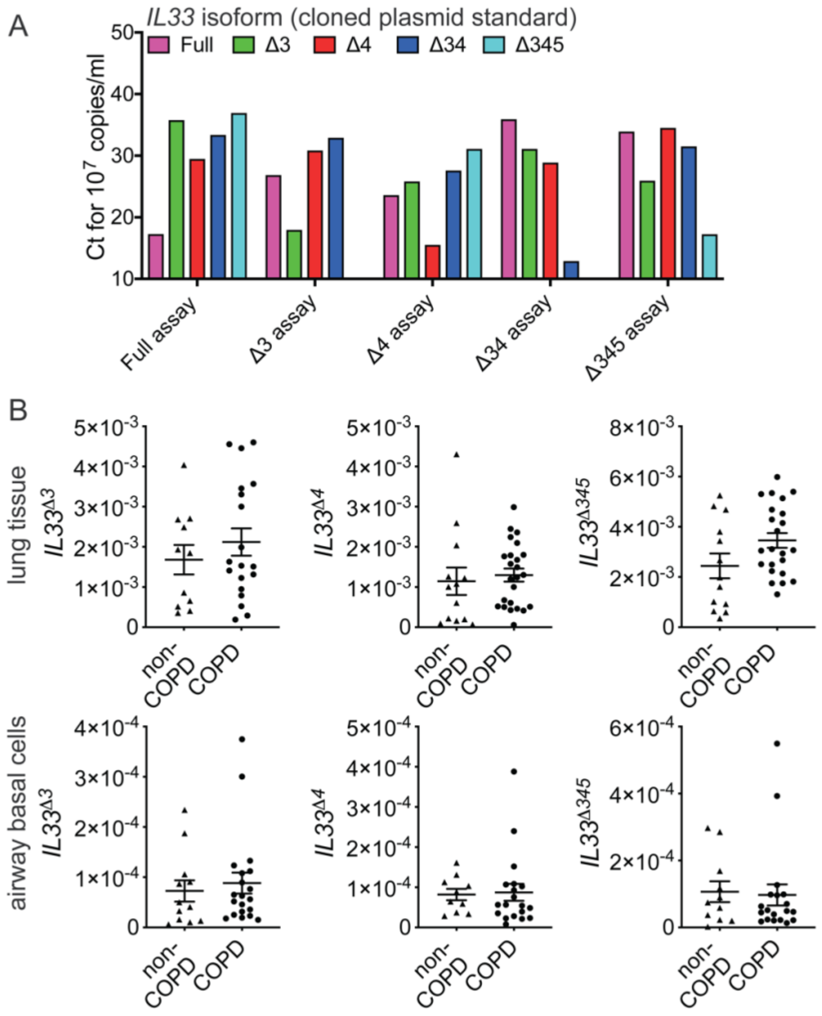

Figure S4. Expression of IL-33 isoforms in COPD and validation of assays, related to Figure 1. A) Validation of isoform-specific qPCR assays by cycling threshold (Ct) measured for each isoform using plasmid standards at 10^7 copies/ml as indicated. Note, while these assays demonstrate high specificity, the efficiency of amplification in complex samples containing multiple IL-33 isoforms is reduced relative to purified single plasmid standards. **B**) Isoform-specific qPCR performed on lung tissue for *IL33*$^{\Delta 3}$ (non-COPD n=11, COPD n=19), *IL33*$^{\Delta 4}$ (non-COPD n=13, COPD n=24) and *IL33*$^{\Delta 345}$ (non-COPD n=13, COPD n=23) isoforms; cultured airway basal cells for for *IL33*$^{\Delta 3}$ (non-COPD n=12, COPD n=20), *IL33*$^{\Delta 4}$ (non-COPD n=10, COPD n=19) and *IL33*$^{\Delta 345}$ (non-COPD n=11, COPD n=19) isoforms, which demonstrate no statistical difference between COPD and non-COPD specimens. Note a specific qPCR assay could not be designed for the *IL33*$^{\Delta 5}$ isoform. Data points displayed with mean ± SEM. Statistical analysis: t-test (B).

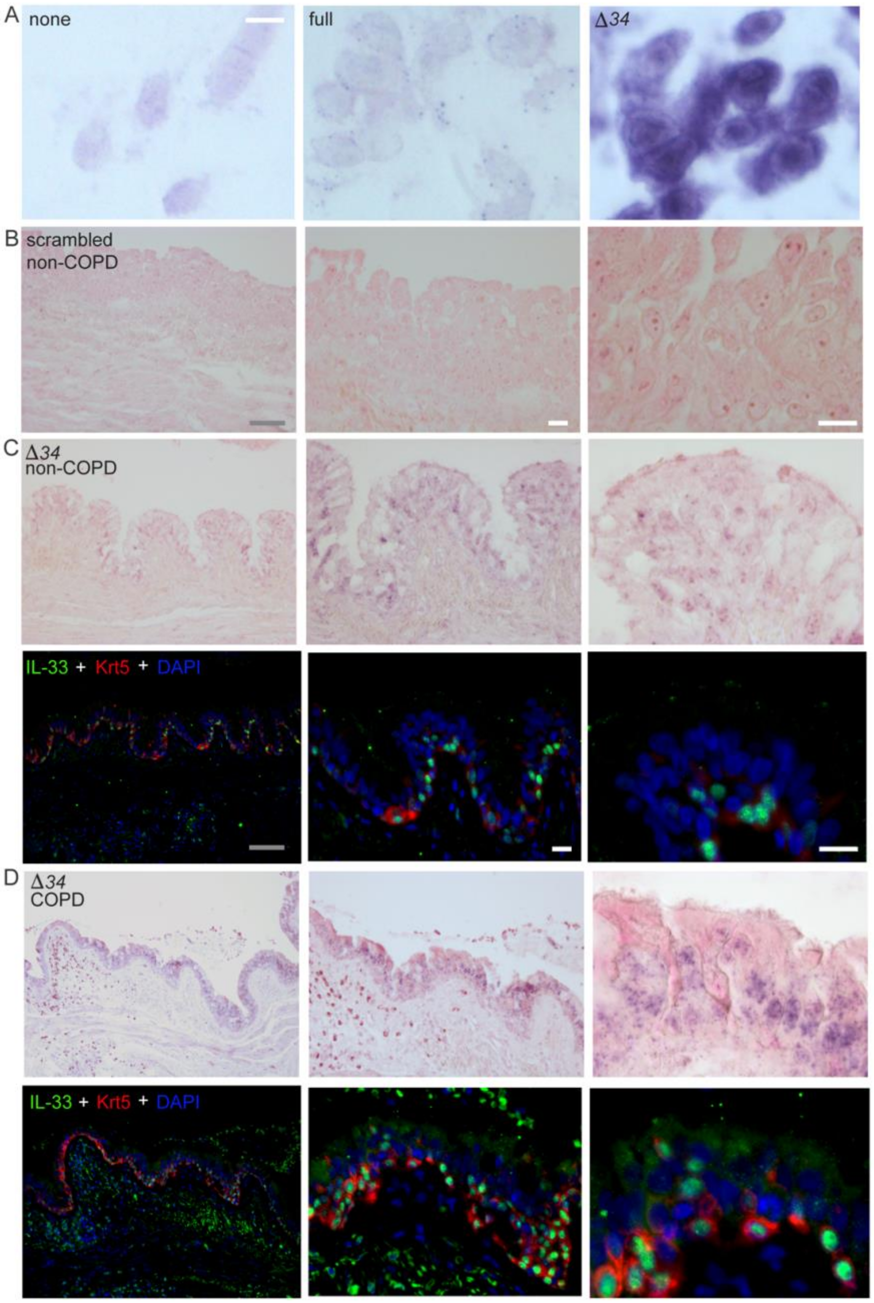

Figure S5. In situ hybridization in COPD and non-COPD tissues with IL-33 protein immunostaining, related to Figure 4. A) Optimized In situ hybridization (ISH) conditions shown for the IL33$^{\Delta34}$ targeting probe in HBE-1 cells overexpressing transcripts as labeled and untransduced HBE-1 cells (none). Probe was detected using alkaline phosphatase conjugated anti-digoxigenin Fab fragments and signal developed with NBT/BCIP substrate (violet) with nuclear fast red counterstain. Scale bar: 10 μm. **B**) ISH performed with scrambled control in non-COPD tissues. Scale bar: 50 μm (gray) and 10 μm (white). **C**) ISH with *IL33*$^{\Delta34}$ targeting probe in non-COPD tissues with corresponding immunofluorescence, anti-IL-33 (green) and Krt5 (red) staining and nuclear counterstain with DAPI, in the same tissue specimen. **D**) ISH staining with *IL33*$^{\Delta34}$ targeting probe in COPD tissue, with corresponding immunofluorescence staining for IL-33 and Krt5 in the same specimen. ISH signal localized primarily to the base of the epithelium, corresponding IL-33 protein staining is seen in nuclear localized and diffusely cytoplasmic patterns.

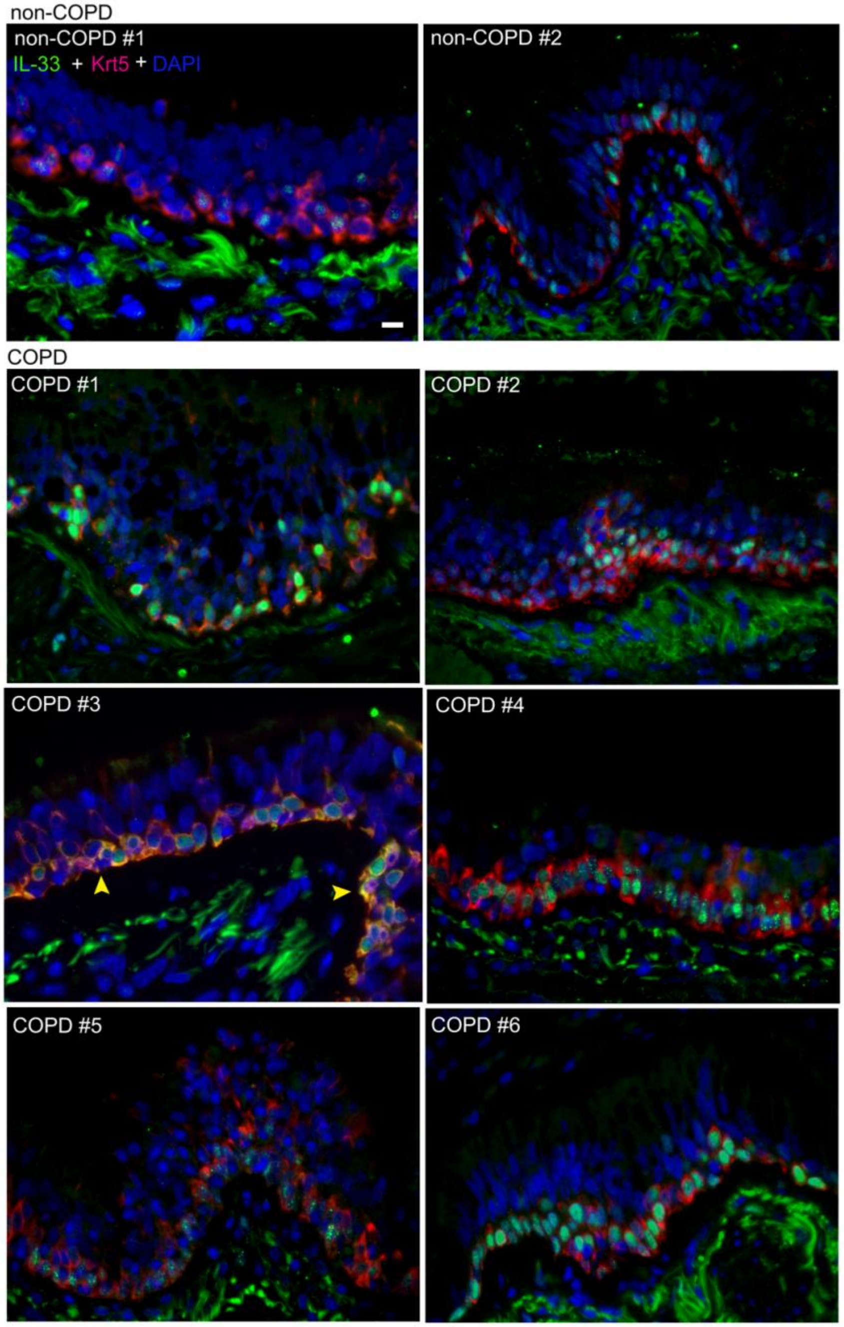

Figure S6. IL-33 immunostaining in COPD and non-COPD tissues, related to Figure 4. Anti-IL-33 and cytokeratin 5 (Krt5) immunofluorescence in non-COPD tissues and COPD tissues for the matched cohort analyzed in Figure 4. Scale bar: 10 μm. Staining demonstrates variable IL-33 staining patterns with localization generally nuclear and lower intensity in non-COPD. In COPD tissues, IL-33 staining is observed varying from nuclear predominant to mixed cytoplasmic/nuclear to diffuse airway staining, with variability observed along length of airway. Krt5 co-staining demonstrates IL-33 is highly expressed within Krt5$^+$ basal cells in all specimens. High background staining of connective tissue was frequently observed in human tissues, and more intense under conditions utilizing 488-conjugated secondary antibodies, under a range of blocking protocols.

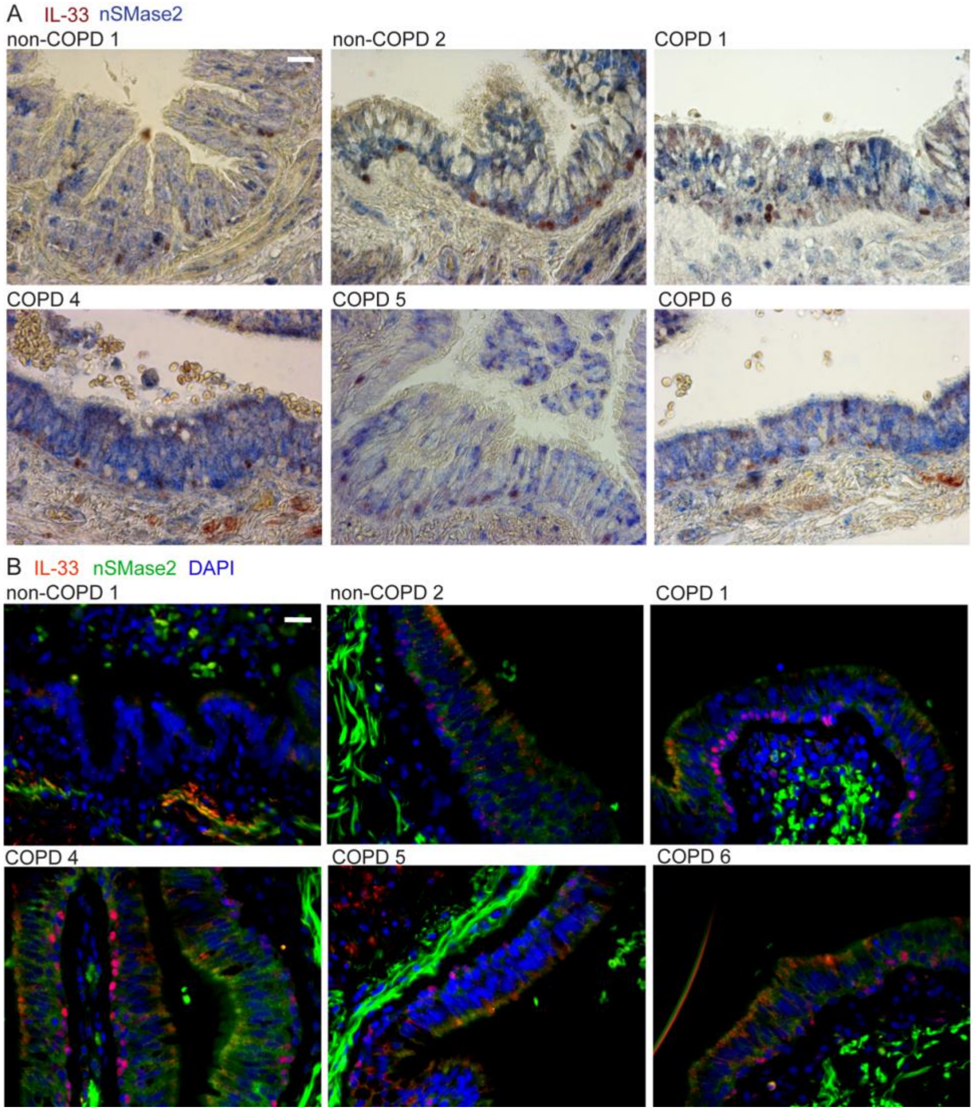

Figure S7. NSMase2 and IL-33 immunostaining in lung tissue, related to Figure 5. A) Anti-IL-33 (AMEC red) and nSMase2 (Vector blue) immunohistochemistry in non-COPD and COPD tissues demonstrating variable staining patterns, with nSMase2 generally more intense and diffuse throughout the airway in COPD relative to non-COPD specimens. Scale bar: 10 μm. B) Anti-IL-33 (red) and nSMase2 (green) immunofluorescence staining in same specimens. Prominent nuclear and variable cytoplasmic IL-33 staining pattern along with diffuse cytoplasmic pan-epithelial nSMase2 staining patterns are noted.

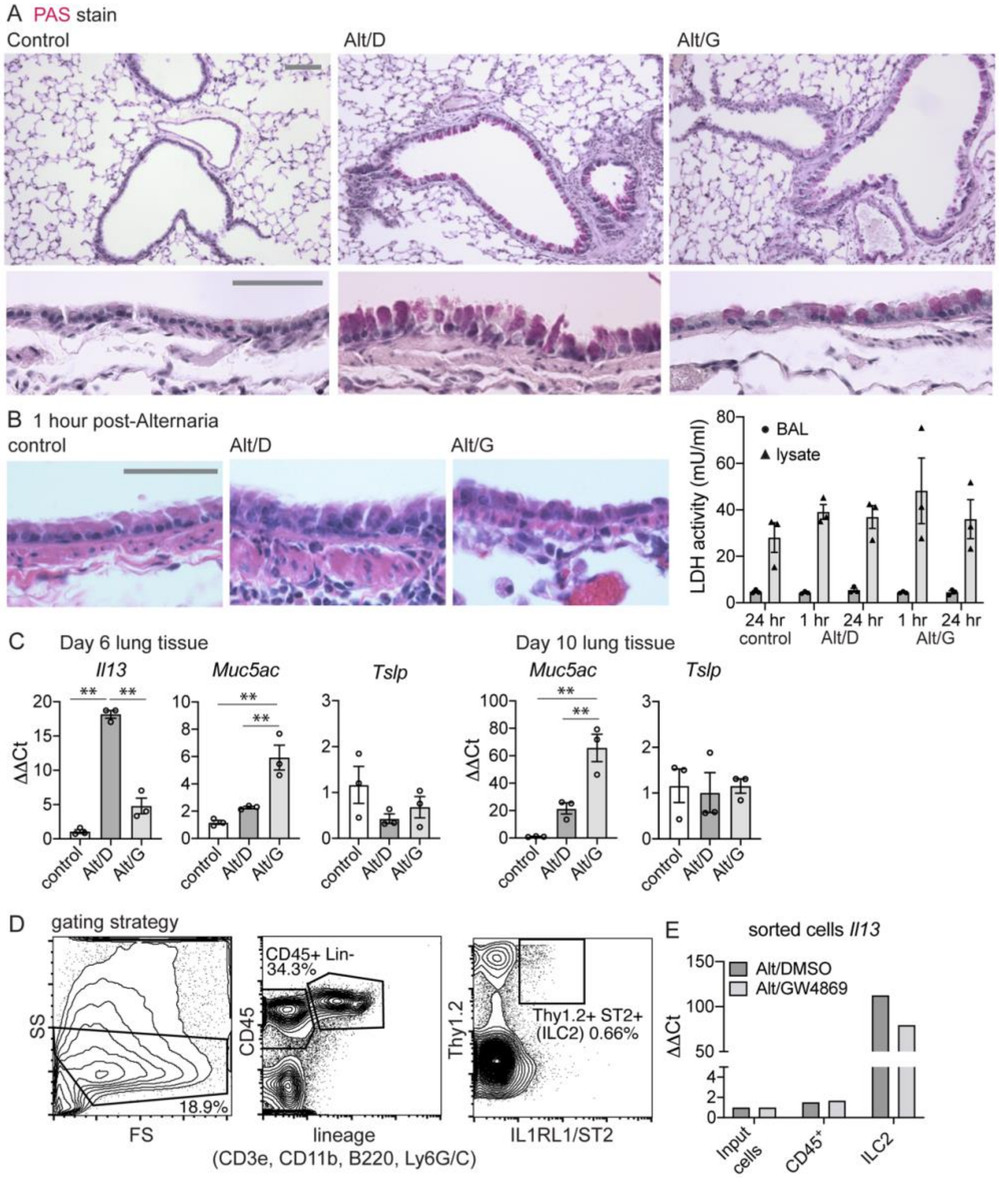

Figure S8. nSMase2 inhibition in *Alternaria* airway disease model, related to Figure 7. A) Periodic Acid Schiff (PAS) staining for mouse tissue sections: PBS (i.n.) only (control), *Alternaria* (i.n.)/2.5% DMSO/PBS (vehicle control, i.p.) (Alt/D) and *Alternaria*(i.n.)/GW4869 (i.p.) (Alt/G) collected at 10 days (24 hrs following the last *Alternaria* treatment). Scale bar: 50 μm. Increased PAS staining is observed in Alt/D and appears partially attenuated in Alt/G. **B**) H&E stained mouse tissue sections collected at 1 hr. post-*Alternaria* treatment, demonstrating intact airway epithelium. Corresponding LDH activity measured in BAL (and tissue lysates for comparison) at 1 hr. and 24 hr. post-*Alternaria* treatment demonstrates no difference between control and *Alternaria* treated BAL samples. Together these results indicate absence of airway cell necrosis with *Alternaria* treatment. **C**) Quantitative PCR for transcripts as indicated at Day 6 and 10 time points demonstrating increased *Il13* and *Muc5ac* in response to Alternaria as early as Day 6, with further increase in both at Day 10. No difference is observed in *Tslp*. **D**) Gating strategy; cells gated on side scatter low populations, then CD45-positive, lineage negative (BD mouse lineage cocktail) and subsequent CD90.2/Thy1.2 and IL-1RL1/ST2 double positive. D) Comparison of *Il13* mRNA expression in total cell input (pooled, n=3), sorted total CD45-positive and sorted ILC2 cell populations demonstrating most abundant signal in ILC2 cells, which was decreased in response to GW4869. Data points displayed with mean $\pm$ SEM. Statistical analysis: one-way ANOVA (C), *P*-values: $^{**}P < 0.01$.
